# Optimised Heterologous Expression and Functional Analysis of the *Yersinia pestis* F1-Capsular Antigen Regulator Caf1R

**DOI:** 10.3390/ijms22189805

**Published:** 2021-09-10

**Authors:** Dharmender K. Gahlot, Gyles Ifill, Sheila MacIntyre

**Affiliations:** 1 School of Biological Sciences, University of Reading, Reading RG6 6UR, UK; ifi6357@mail.ubc.ca; 2 Department of Molecular Biology, Umeå University, 901 87 Umeå, Sweden; 3 Umeå Centre for Microbial Research (UCMR), Umeå University, 901 87 Umeå, Sweden; 4 Department of Microbiology and Immunology, University of British Columbia, Vancouver, BC V6T 1Z3, Canada

**Keywords:** *Yersinia pestis*, F1 capsule, transcriptional regulator, Caf1R, functional analysis

## Abstract

The bacterial pathogen, *Yersinia pestis*, has caused three historic pandemics and continues to cause small outbreaks worldwide. During infection, *Y. pestis* assembles a capsule-like protective coat of thin fibres of Caf1 subunits. This F1 capsular antigen has attracted much attention due to its clinical value in plague diagnostics and anti-plague vaccine development. Expression of F1 is tightly regulated by a transcriptional activator, Caf1R, of the AraC/XylS family, proteins notoriously prone to aggregation. Here, we have optimised the recombinant expression of soluble Caf1R. Expression from the native and synthetic codon-optimised *caf1R* cloned in three different expression plasmids was examined in a library of *E. coli* host strains. The functionality of His-tagged Caf1R was demonstrated in vivo, but insolubility was a problem with overproduction. High levels of soluble MBP-Caf1R were produced from codon optimised *caf1R*. Transcriptional-*lacZ* reporter fusions defined the P_M_ promoter and Caf1R binding site responsible for transcription of the *cafMA1* operon. Use of the identified Caf1R binding *caf* DNA sequence in an electrophoretic mobility shift assay (EMSA) confirmed correct folding and functionality of the Caf1R DNA-binding domain in recombinant MBP-Caf1R. Availability of functional recombinant Caf1R will be a valuable tool to elucidate control of expression of F1 and Caf1R-regulated pathophysiology of *Y. pestis*.

## 1. Introduction

*Yersinia pestis* is a deadly zoonotic bacterial pathogen. It has killed around 200 million people in three major global plague pandemics and continues to be a threat to this day with endemic outbreaks and sporadic cases [1,2]. An intensively studied virulence factor of *Y. pestis* is the ‘capsular’ antigen known as F1 [3,4,5,6]. Structurally, F1 is composed of thin linear polymers of Caf1 protein subunits, assembled on the bacterial cell-surface via a cognate periplasmic chaperone (Caf1M) and an outer membrane usher (Caf1A), which then collapse on the surface to form a capsule-like structure [4,5,7,8]. Production of F1 is induced during infection following transmission from the flea vector to humans or to a rodent reservoir [9,10]. This surface capsule-like structure helps bacteria neutralise a robust immune response by conferring antiphagocytic ability [10]. The F1 capsule also has substantial applied value. Due to its surface location, high level of expression and the fact that the F1 polymer is unique to *Y. pestis*, F1 remains a primary target for plague diagnostics [11,12,13,14] and a key component of anti-plague vaccines [6,15,16,17].

Details of the mechanism of expression of F1 is less well understood. Expression from the *caf* locus is controlled by a transcriptional activator known as Caf1R [18]. Caf1R belongs to the AraC/XylS (A/X) family of bacterial transcriptional regulators [19,20]. Regulators belonging to the A/X family are widespread among both Gram-negative and Gram-positive bacteria, spanning >80% of the ‘non-redundant’ prokaryote genomes [21]. They control expression of genes involved in diverse biological processes from metabolism to stress responses and virulence, as reviewed by Cortes-Avalos et al. [19,20]. Proteins of this family are characterised by a signatory DNA-binding domain (DBD) of ~100 amino acids, often located at the C-terminus, accompanied by a sensing or oligomerisation domain of ~100–200 amino acids that could be located at either end, N- or C-terminus. A few regulators of this family possess only the signatory DBD, such as MarA and its homologue, SoxS [22]. The signatory DBD contains a conserved tertiary structure composed of 7 α-helices and binds to the promoter/operator region of target operon(s) via two Helix-turn-Helix (HTH) motifs, connected by a central α-helix or turn [19,23]. The A/X family regulators often activate expression although some such as AraC may act as both activator and repressor [19,20].

Bacterial regulators are generally expressed at low levels in the cell and many proteins of this family when overexpressed, tend to form ‘inactive’ aggregates also known as inclusion bodies. This is a likely explanation why only a small number (*n* = 126), have been characterised experimentally so far [19], as it is therefore inherently difficult to purify A/X family members in the soluble ‘active’ form. MarA and Rob are the only two proteins of A/X family whose high-resolution crystal structures have been deduced with their cognate promoter DNA ligands [24,25]. Availability of these structures has helped to elucidate the mode of action of these regulators and interaction with respective DNA binding sequences. Several approaches are commonly used to improve recombinant expression of problematic proteins in *Escherichia coli* [26,27,28]. There are a number of examples of successful purification of A/X family regulators [24,29,30,31,32,33,34] using either a small-peptide His_6_-tag or the bulky protein-tag, maltose binding protein (MBP), which has been proven to enhance solubility of fusion partners [35].

Given the central regulatory role of Caf1R to control expression of the F1 capsular antigen of this deadly pathogen, the ability to purify soluble active Caf1R would greatly facilitate study of the regulatory mechanism of the *caf* locus. Examples of current methods where overexpressed functional Caf1R might be used to elucidate its activity include DNase foot-printing, electrophoretic mobility shift assay (EMSA), ChipSeq, antibody pull- down assays and structure determination [24,25,30,36,37,38,39]. In depth study of the structure and regulatory mechanisms of Caf1R would also provide opportunities to extend general understanding of A/X family regulators. Therefore, with so little known about regulation of the *caf* locus, in contrast to the wealth of data available on structure, assembly and vaccine potential of the F1 capsular antigen itself, this study has focused on assessing different expression systems for recombinant production of functional Caf1R in *E. coli.* Native and synthetic ‘codon-optimised’ *caf1R* alleles were cloned downstream of either the small, His_6_-tag or the bulky MBP-tag [35]. Three expression vectors were trialled, pBADHisA, pET28a+ and pMALc2x, and the expression level of recombinant Caf1R was monitored in a library of *E. coli* host strains. Production of high levels of soluble Caf1R from a codon-optimised *caf1R* allele tagged with MBP (MBP-Caf1R*) was achieved, and was most successful when expressed from the protease deficient strain *E. coli* K12 ER2508. In order to demonstrate functionality, both P_M_ promoter and Caf1R-binding *caf* DNA motif, upstream of the *cafMA1* operon, were first identified using promoter fusion studies. This cognate Caf1R binding DNA sequence was subsequently used in EMSA to demonstrate functional binding of the MBP-Caf1R* fusion protein.

## 2. Results

### 2.1. An Overview of Caf1R 

**Figure 1:**
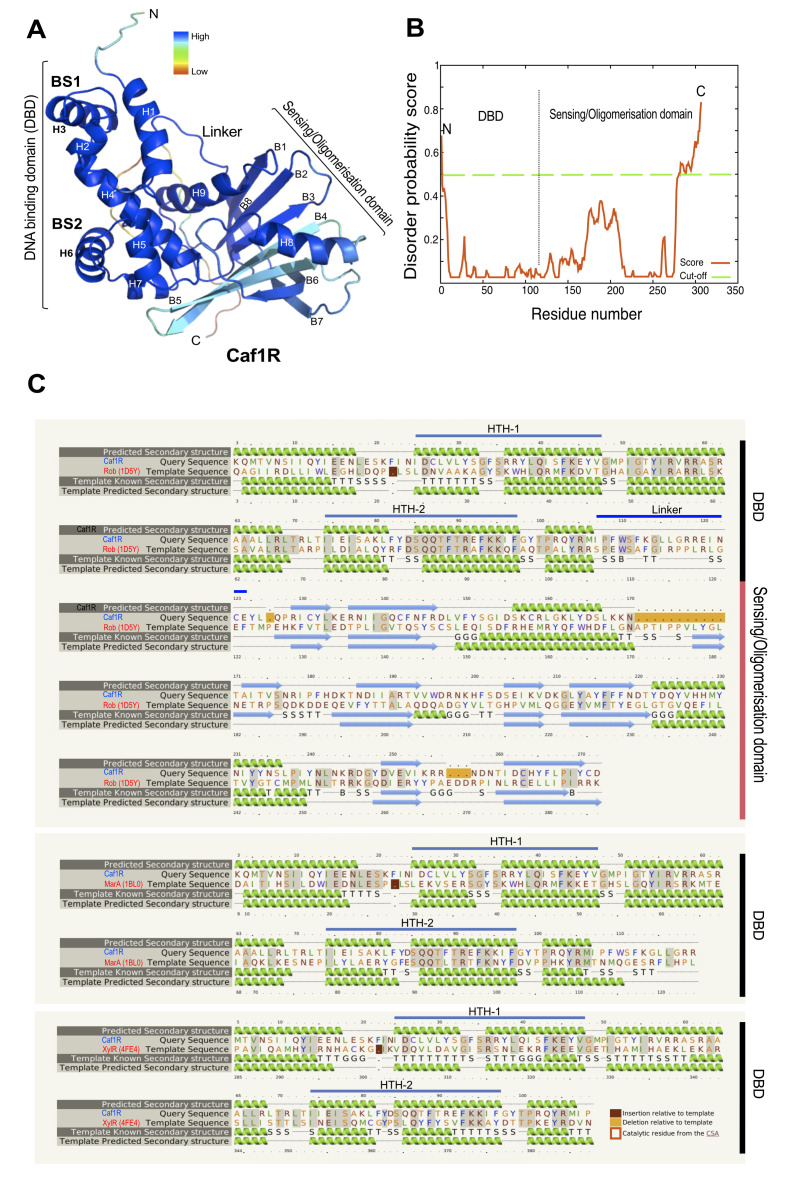
Caf1R modelled structure and its homology with *E. coli* Rob, MarA and XylR. (**A**) IntFOLD3.0 [49] predicted model of the full-length Caf1R on Rob protein template (PDB 1D5Y). Quality of prediction is indicated by the β-factor colour intensity and its ≥70% non-redundancy, computed by IntFOLD3.0. Predicted DNA-binding domain (DBD) at the N-terminus composed of seven alpha helices (H1–H7), connected by an unstructured linker to the putative sensing/oligomerisation domain at the C-terminus, comprised of H8–H9 and eight β-strands (B1–B8). (**B**) Disorder prediction plot (per residue) of the modelled structure, reinforces reliability of the Caf1R model. MacPyMOL1.3 was used to visualise and generate the ribbon diagrams of modelled Caf1R. (**C**) Phyre2 [50] predicted secondary structures alignment of the full-length native Caf1R (query) with the closest A/X family structural homologues, Rob (PDB 1D5Y), MarA (PDB 1BL0) and XylR (PDB 4FE4) as indicated. The helix-turn-helix (HTH) motifs of Caf1R-DBD, HTH-1 (H2–H3) and HTH-2 (H5–H6) are displayed with a thick blue line over respective helices. Amino acids highlighted in grey indicate aa identity of corresponding protein template with Caf1R: Rob (24%), MarA (34%) and XylR (35%). Seamless alignment of the Caf1R predicted secondary structures on the Rob template, confirms reliability of full-length Caf1R model.

Bacterial proteins with a PROSITE scan score of 12.52–30.74 are considered a member of DNA-binding transcriptional regulators of the extensively dispersed AraC/Xyls (A/X) family (PROSITE ID PS01124). With a score of 28.81, Caf1R is defined as a member of this family. The theoretical pI of Caf1R is computed to be 9.17, indicating a highly basic protein. There are 32 of the negatively charged amino acids, Asp and Glu, and 43 of the positively charged amino acids, Arg and Lys, consistent with it being a positively charged protein at pH 7. The instability index of Caf1R is computed to be 50.34 classifying Caf1R as a relatively unstable protein. Analysis of Caf1R with Pfam (http://pfam.xfam.org/protein/, accessed on 18 March 2021), validates Caf1R as a two-domain protein, with a signatory DNA-binding domain (DBD) at the N-terminus, encompassing residues 1–106 and a putative GyrI-like small molecule binding domain at the C-terminus, from residue 125–272. Both domains are connected by a flexible linker (Figure 1). The two-characteristic helix-turn-helix (HTH) motifs, of the DBD domain lie within residues 25–47 (HTH-1) and 74–97 (HTH-2) (Figure 1). The existence of a putative GyrI-like small molecule binding domain at the C-terminus suggests the possibility of Caf1R responsiveness to some as yet unidentified small molecule(s) as has been demonstrated for other A/X regulators possessing a sensing /oligomerisation domain. Examples include: carbohydrate responsive AraC [40], RhaS and RhaR [41], XylR [30], and XylS [42]; urea responsive, UreR [43]; bile salts and fatty acid responsive, ToxT [44,45], Rob [46]; and exclusively fatty acids responsive, HilD, Rns, and VirF [47,48].

To date, MarA and Rob are the only A/X family members for which the high-resolution crystal structures have been deduced in complex with DNA [24,25]. These structures have aided significantly in elucidating the activity of these regulators. Recently, XylR was also overexpressed as a XylR-His_6_ tagged protein, purified and the crystal structure solved [30]. These proteins share about 90% (Rob, 25% aa identity), 94% (MarA-DBD only; 34% aa identity) and >95% (XylR, 35% aa identity with DBD) structural homology with predicted Caf1R structure (Figure 1). 

### 2.2. Caf1R Expression from the Native Gene, Cloned in pBADHis_6_ Plasmid 

**Figure 2:**
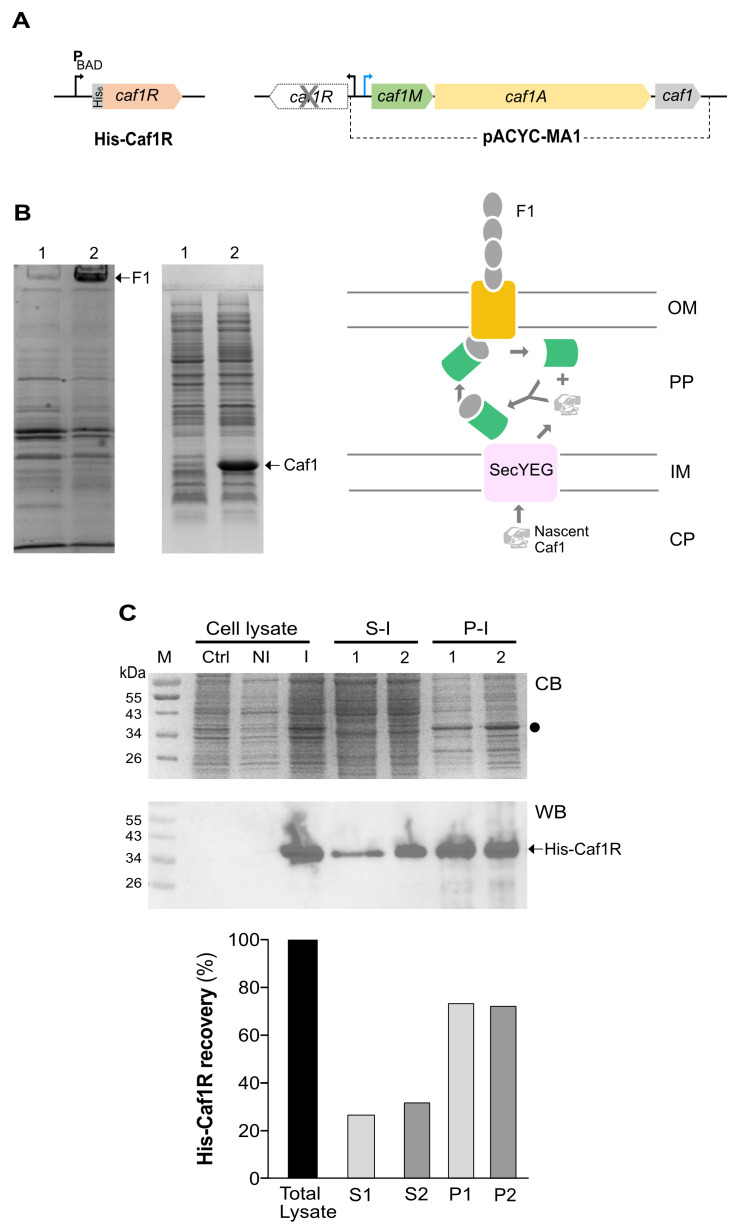
His-Caf1R expression (native *caf1R*) from *E. coli* Top10. (**A**) Schematic of His-Caf1R expressed from pBADhCaf1R and genetic organisation of the *caf* locus in pACYC-MA1 (**B**) Confirmation of functionality of His-Caf1R. Following 4 h induction with 0.02% L-arabinose, surface Caf1 polymer (F1) was extracted from *E. coli* Top10 cells transformed with pBAD vector + pACYC-MA1 (lane 1) or with pBADhCaf1R + pACYC-MA1 (lane 2) and processed for SDS-PAGE, as described in methods. Coomassie blue stained SDS-PAG of surface extracted F1 polymer (applied to gel without heating) and the heat-denatured Caf1 subunits are shown. Diagram depicts Chaperone-usher pathway of Caf1 subunit assembly into polymeric fibres of the F1 capsule [4,7,8,51]. Green (Caf1M chaperone) Yellow (OM usher) Grey (Caf1 subunit), OM (outer membrane), IM (inner membrane), PP (periplasm), CP (cytoplasm). (**C**) Solubility analysis of His-Caf1R, CB stained SDS-PAG and WB of respective samples. Cell lysates of indicated samples were prepared by mixing cells from 50 mL culture in 5 mL of lysis buffer (S1/P1 in 20 mM NaPO_4_, 500 mM NaCl, 3 mM DTT, 10% glycerol; pH 7.4 and S2/P2 in 20 mM Tris-HCl, 200 mM KCl; pH 7.4) supplemented with 1× EDTA-free protease inhibitor followed by sonication. S and P fractions were isolated by ultracentrifugation of sonicated cell lysates. NI (non-induced), I (0.02% L-arabinose induced at 37 °C-4 h). Cell lysates were analysed by mixing into 4× SDS-PAGE sample buffer. Expected location of His-Caf1R is represented by a dot (●). Expression of His-Caf1R was identified with antiHis-HRP monoclonal antibody. M, protein size (kDa) marker.

A characteristic feature of recombinant expression of A/X family regulators is insolubility. Therefore, to ensure tight control of expression, a P_BAD_ promoter-based plasmid, pBADhCaf1R (Figure S1), expressing N-terminal His_6_-tagged Caf1R was initially tested for expression of His-Caf1R in *E. coli* Top10 cells. Functionality of this His-Caf1R construct was confirmed in vivo using a trans-complementation system with Caf1R regulator expressed from pBADhCaf1R and the *cafMA1* locus plus intergenic regulatory region located on a second plasmid, pACYC-MA1 (Figure 2A). The pACYC-MA1 encodes all components required for assembly of F1 capsule, the assembly components (Caf1M chaperone, Caf1A usher) as well as the F1 structural subunit Caf1, but no Caf1R regulator (Figure 2A). *E. coli* cells transformed with pBADhCaf1R + pACYC-MA1 and induced at mid-logarithmic growth with 0.02% L-arabinose for 4 h at 37 °C, expressed large amounts of F1. This was visible on CB stained SDS-PAGs of surface extracted F1 capsule and the heat-denatured Caf1 subunit [15.5 kDa] (Figure 2B, lane 2). No F1 capsule and Caf1 subunit were visible from *E. coli* cells transformed with the empty pBAD vector + pACYC-MA1 (Figure 2B, lane 1), confirming the ability of the recombinant His-Caf1R to function effectively in vivo as an activator of the *caf* locus.

To monitor solubility of His-Caf1R, a 50 mL culture of recombinant *E. coli* Top10/pBADhCaf1R was induced with 0.02% L-arabinose at 37 °C for 4 h. The soluble and insoluble fractions of His-Caf1R, isolated by ultracentrifugation at 50,000 rpm for 1 h, were analysed by CB staining and immunoblot (Figure 2C). A potential protein band of His-Caf1R size [40.98 kDa] observed by CB staining only in the insoluble (P) fractions appeared to be partially masked by a host protein. The immunoblot confirmed presence of His-Caf1R in both P and S fractions, with a higher level in the insoluble pellet fractions (Figure 2C). Conclusively, these results indicate that while the low level of expression of soluble His-Caf1R was functional, the level of recovery of soluble regulator was far too low for purification of sufficient levels for in vitro functional studies. It was proposed that alteration of expression plasmids and *E. coli* host strains could enhance both the level and solubility of His-Caf1R. Hence, the frequently used T7-promoter based expression system that had been used for the overexpression and purification of the A/X family regulators, Rob [24,29], XylR [30] and TetD [31], was tested to optimise His-Caf1R overexpression.

### 2.3. Caf1R Expression from the Native Gene, Cloned in pET28a^+^ Plasmid

To elevate expression of His-Caf1R, ideally without impairing solubility, *caf1R* was cloned downstream of the His_6_-tag and thrombin cleavage coding sequences in the pET28a^+^ expression vector. This construct, designated pEThCaf1R plasmid, expresses native His-Caf1R under control of the T7-promoter (Figure S2). Recombinant expression of His-Caf1R was monitored from the classical *E. coli* BL21(DE3) host strain at 37 °C with 1.0 mM IPTG and 4 h induction (Figure S3). While immunoblotting confirmed recovery of His-Caf1R in both the soluble (S) and insoluble (P) fractions, a host protein (~40 kDa) masked any stainable His-Caf1R in both fractions. Significantly, an intense band at ~14 kDa in the P fraction, also indicated truncation of the His-Caf1R to produce an insoluble His_6_-tagged N-terminal DBD of His-Caf1R (calculated size, 16.45 kDa). Induction at lower temperature and IPTG concentration [28 °C and 0.1 mM IPTG for 16 h] did not improve recovery of intact His-Caf1R. A major proportion of His-Caf1R was still truncated and formed inactive aggregates, as seen on the immunoblot (Figure S3). Thus, expression of His-Caf1R from the T7 promoter resulted not only in low levels of stainable product, but also in truncation of His-Caf1R and recovery primarily in the insoluble fraction. 

A possible explanation for the truncation is that the C-terminal domain of Caf1R, which putatively acts as a sensing or oligomerisation domain (Figure 1), leads to aggregation and cleavage releasing the N-terminal His-tagged DBD. There are seven identified Cys residues, C27, C123, C131, C142, C158, C263 and C271 in the coding sequence of Caf1R (Figure 1C). Only one of which is in the DBD (C27). The remaining six are in the putative C-terminal sensing/oligomerisation domain (Figure 1C), and could result in misfolding and/or stability issues. Moreover, the number of Cys residues is quite unusual for A/X regulators and the DBD of Rob, MarA and SoxS do not contain any Cys residue (Figure 1). This hypothesis is supported by the results from others where individual domains of X/R regulators were purified separately in more soluble and active form than that of entire protein [52,53,54]. In addition, those A/X regulators that lack a sensing/oligomerisation domain, for example MarA and its homologue SoxS have been successfully over-expressed and functionally characterised [22,55]. 

### 2.4. Recombinant Expression of Caf1R from the Codon-Optimised caf1R Gene in pET28a+ Plasmid

A recognised difficulty encountered during overexpression of low copy number proteins relates to inherent suboptimal codons in the open reading frame (ORF). If an ORF contains rare or suboptimal codons, not often used by *E. coli*, then overexpression of that protein in *E. coli* would be severely diminished to a point of being undetectable [27,28,56]. In particular, the codons for Arg (R) (AGG, AGA, and CGA), Leu (L) (CTA), Ile (I) (ATA) and Pro (P) (CCC) can be problematic particularly if two of these codons are present together or in a tandem repeat [27,28,56]. Analysis of the *caf1R* ORF by rare codon finder, RaCC tool (http://nihserver.mbi.ucla.edu/RACC/, accessed on 18 March 2021), identified 32 rare codons: 18 Arg (R), 10 Ile (I), 3 Leu (L) and 1 Pro (P) (Figure S4). Among these detected rare codons, 8 Arg and 3 Ile create tandem double repeats and thus could be responsible for the low expression level of recombinant Caf1R from the native gene as suggested by Rosano and Ceccarelli [27,28,56]. This distribution of rare codons throughout *caf1R* ORF was further validated by another rare codon analysis tool (GenScript, Piscataway, NJ, USA). Additionally, the codon usage frequency for the expression of native *caf1R* in an *E. coli* host was predicted to be 0.6, about half of the recommended, 1.0 (GenScript, Piscataway, NJ, USA). 

**Figure 3:**
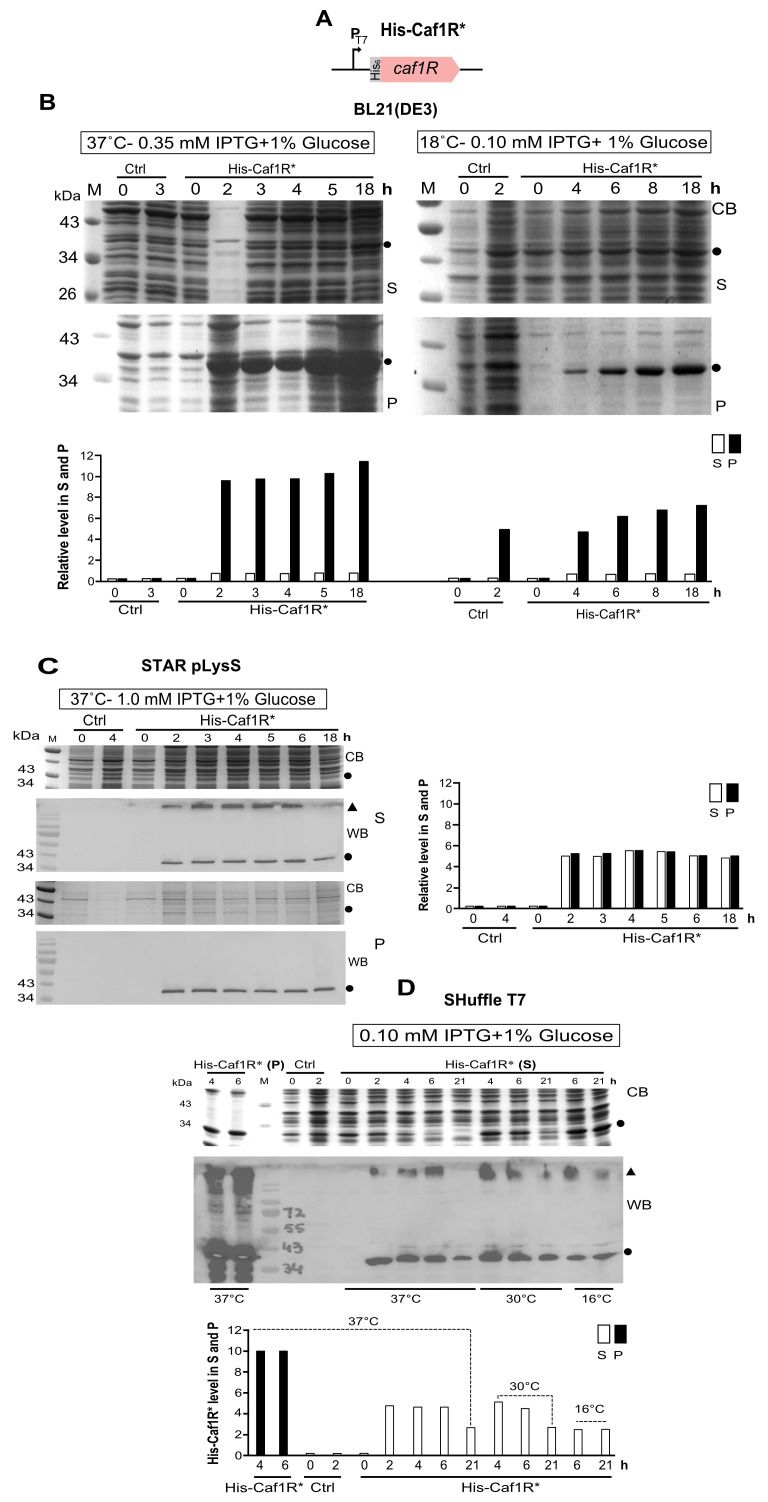
Expression of His-Caf1R* (codon-optimised *caf1R*) from *E. coli* BL21(DE3) and its variants. (**A**) Schematic of His-Caf1R* from pEThCaf1R* plasmid. Soluble (S) and insoluble (P) fractions of His-Caf1R from *E. coli* BL21(DE3) (**B**) and its two variants, STAR pLysS (**C**) and SHuffle (**D**) were prepared after specified IPTG-induction at 37 °C, 30 °C, 18 °C and 16 °C. The S and P fractions were prepared by mixing an equal number of cells [1 OD unit] in 100 μL of Bugbuster master mix followed by centrifugation [20,000 rpm–15 min/4 °C]. Expected location of full-length His-Caf1R is indicated by a dot (●) and high-molecular weight insoluble aggregates is represented by a black triangle (Δ). Expression of His-Caf1R* is validated by western immunoblotting with anti-His-HRP monoclonal antibody, and comparison with negative control, Ctrl (corresponding strain containing empty plasmid, pET28a^+^). CB- Coomassie blue stained SDS-PAGE; non-induced samples [0 h] and M, protein size (kDa) marker. Relative level of total His-Caf1R in S and P fractions of respective strain was quantified, from CB-stained SDS-PAGE (**B**) and from western blot (**C**,**D**).

A codon-optimised caf1R* (Figure S4) was designed and inserted into pET28a+ plasmid, to create pEThCaf1R*, expressing synthetic codon-optimised caf1R* under control of the T7 promoter (Figure S5). Expression of the codon-optimised His-tagged Caf1R (His-Caf1R*) was monitored in *E. coli* BL21(DE3) and two derivative strains: STAR pLysS (to enhance *caf1R* mRNA stability with low *E. coli* protein background) and SHuffle T7 (to enhance DsbC-mediated cytoplasmic disulphide bond formation). Expression was initially monitored at 37°C with 0.35 mM IPTG induction at 1.5, 3.0, 4.5, 6.0 and 18 h post-induction by SDS-PAGE and WB. No protein band was detected at the expected location of His-Caf1R (~38.5 kDa). This was attributed to IPTG-mediated substrate toxicity due to very high level of expression [57]. To rectify this toxicity, cells containing codon-optimised recombinant Caf1R plasmid were routinely grown in the presence of glucose irrespective of induction. Expression of His-Caf1R was reassessed using the same *E. coli* strains and induction with IPTG + 1% glucose (Figure 3). With native BL21(DE3) and induction at 37 °C, a prominent protein band, corresponding to the expected location of His-Caf1R*, was detected in the pellet fractions of induced cells. This was at least 10-fold more abundant than soluble His-Caf1R* which could not be clearly identified in the soluble fraction (Figure 3B). A decrease in both induction temperature and IPTG-concentration, to 18 °C and 0.10 mM respectively, did not markedly enhance solubility-a major prominent band of His-Caf1R* was still detected in the insoluble pellet fractions (Figure 3B). With STAR pLysS cells tight control of expression was observed irrespective of length of induction there was only a low level of recovery of full-length His-Caf1R* in both soluble and insoluble fractions (Figure 3C). While expression in the SHuffle T7 strain was significantly higher, His-Caf1R* aggregates were evident irrespective of temperature in both S and P fractions, identifiable by immunoblot (Figure 3D). High recovery in the pellet indicated that enhanced cytoplasmic disulphide bond formation did not improve solubility of the C-terminal domain of Caf1R. From these results it can be concluded that the codon-optimised caf1R* is producing much higher levels of protein than native *caf1R*, as synthetic His-Caf1R* was detected very easily by CB-staining in the pellet fractions, when expressed from native BL21(DE3) (Figure 3B). Despite the enhanced level of recombinant Caf1R*, the problem of solubility still remained, indicating potential value of use of a solubility enhancing vector.

### 2.5. Caf1R Expression from the Codon-Optimised Gene, Cloned in pMALc2x Plasmid

Because use of the small, cleavable His_6_-tag did not produce the required level of soluble recombinant Caf1R, this suggested that one of the commonly used solubility enhancer bulky-tags, maltose binding protein (MBP) [35] may be more suitable to improve recovery of soluble Caf1R for subsequent functional assays. The MBP-tag has been used in overexpression and purification of three other A/X family regulators, SoxS [32], Rns [33] and PchR [34]. Hence, plasmids were constructed with full-length native *caf1R* or codon-optimised caf1R* fused downstream of *malE* in the pMALc2x plasmid under the control of the IPTG-inducible P*tac* promoter. These plasmids were designated, pMALCaf1R (Figure S6) and pMALCaf1R* (Figure S7), respectively. Initial studies with pMALCaf1R revealed instability of the soluble fusion construct when expressed in *E. coli* BL21 (DE3) (Figure S8). Therefore, in an effort to retain intact MBP-Caf1R fusion and enhanced solubility, the ATP-dependent proteases deficient *E. coli* K12 strain ER2508 was used as host with a marked positive effect on recovery and solubility of MBP-Caf1R (Figure S8).

**Figure 4:**
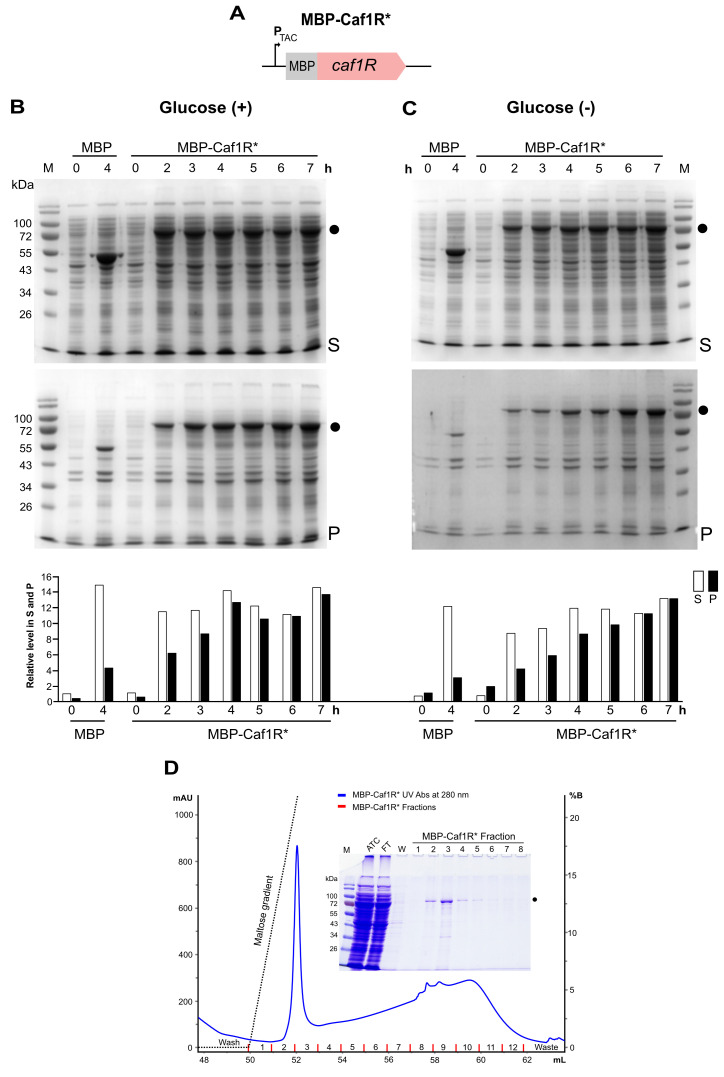
Expression of MBP-Caf1R* (codon-optimised *caf1R*) from *E. coli* K12 ER2508. (**A**) Schematic of MBP-Caf1R* from pMALCaf1R* plasmid. (**B**,**C**) CB-stained SDS-PAGE analysis and quantitation of MBP-Caf1R*. Cultures were induced at 37 °C with 0.35 mM IPTG ± glucose (1%) and sampled over 2–7 h, as indicated. Soluble supernatant (S) and insoluble pellet (P) fractions were prepared from an equal number of cells [1 OD unit], lysed in 100 μL MBP-TrapHP column buffer with 1× protease inhibitor (Roche) followed by centrifugation at 20,000 rpm/15 min at 4 °C. Pellets were resuspended in 100 μL buffer. Dot (●), full-length MBP-Caf1R, M, protein size (kDa) marker. Non-induced samples [0 h]; Ctrl, cells expressing empty plasmid, pMALc2x. Relative level of MBP-Caf1R* in S and P fractions of ±glucose samples was quantified. (**D**) Elution profile of MBP-Caf1R* from MBP-TrapHP column, from culture induced at 25 °C for 5 h, see Methods for detail. The SDS-PAGE profile of fractions 1–8 is shown. ATC-sample applied to the column, FT-flow through and W-wash fraction. M, protein size (kDa) marker.

The results of expression of codon-optimised MBP-Caf1R* fusion in *E. coli* K12 E2508 with 0.35 mM IPTG ± 1% glucose at 37 °C (Figure 4B,C) were significantly higher even than for MBP-Caf1R. A major band correlating to MBP-Caf1R* (~78.98 kDa) was detectable (by CB staining) in all induced samples (Figure 4B,C) and validated by immunoblotting (data not shown). Importantly, the majority of MBP-Caf1R* was now recovered in the soluble supernatant fraction. Following amylose affinity chromatography as described in Methods, successful recovery of soluble MBP-Caf1R* was purified (Figure 4D). The peak fraction (Fraction 3) had a protein concentration of ~0.38 mg/mL. 

While the presence of glucose in the growth medium resulted in higher levels of MBP-Caf1R*, higher levels of background host proteins were also produced (Figure 4B). Additionally, as culture incubation time was prolonged, the relative ratio of MBP-Caf1R* in the insoluble pellet fractions also increased (Figure 4B,C). Hence, to maximise recovery of soluble and stable MBP-Caf1R*, and to minimise contamination from host background proteins, we suggest, for incubations at 37 °C, that induction in the absence of glucose for a maximum of 3–5 h induction time would be most suitable for large scale production and purification of soluble MBP-Caf1R*.

### 2.6. In Vivo and In Vitro Functional Analysis of Caf1R

A DNA-Protein binding assay (EMSA) was used to assess the functionality of the recombinant fusion protein, MBP-Caf1R*. Prior to performing this, it was crucial to accurately identify the Caf1R binding site and promoter involved in activation of transcription of the *caf* locus. These were identified using progressively shorter promoter-*lacZ* reporter fusions within the intergenic regulatory region upstream of the *cafMA1* operon, with and without plasmid mediated complementation of Caf1R. 

### 2.7. Identification of Caf1R-Regulated Promoter(s) of the Caf Locus

**Figure 5:**
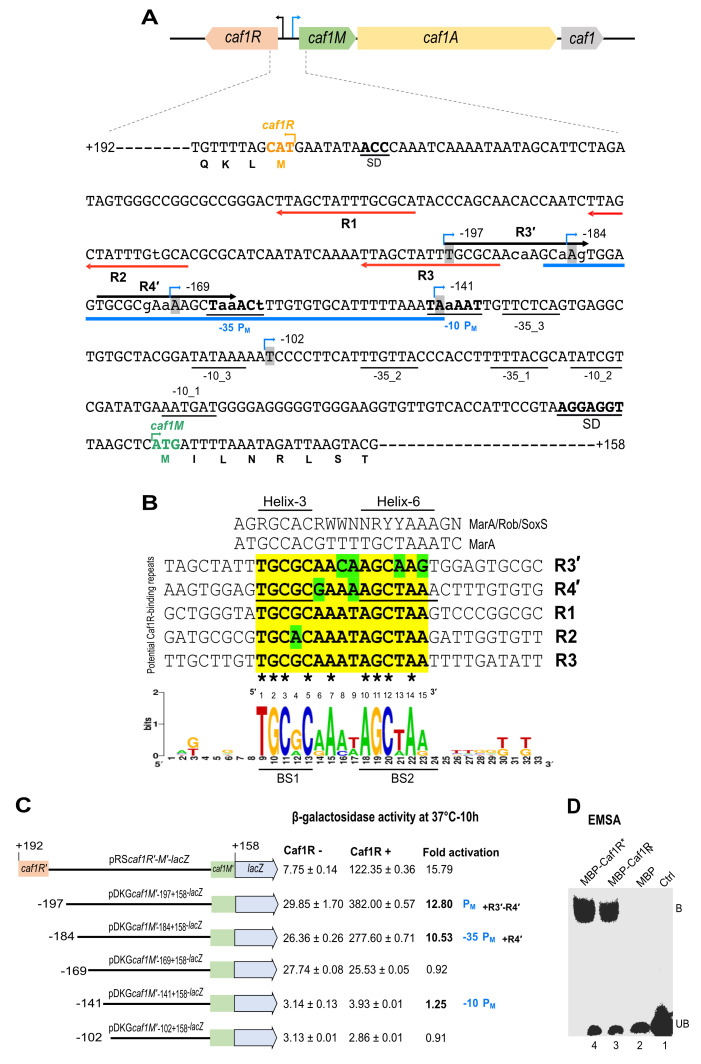
Caf1R facilitates transcription activation of the *caf* locus. (**A**) Organisation of genes of the *caf* locus [5.128 kb]. Genes are drawn as per their size and location; orientation of transcription is indicated, *caf1R* (black arrow) and *cafMA1* (blue arrow). Sequence encompassing *caf1R-cafMA1* intergenic region illustrates regulatory features. The *caf1R* and *cafMA1* Shine-Dalgarno (SD) motifs are as predicted [18,61,62]. For clarity, σ70 bacterial promoter elements (-10 and -35) only for the *cafMA1* operon are indicated. Caf1R-dependent promoter, P_M_ regulating *cafMA1* operon is confirmed in this study. P_M_ consensus elements (bold capital nt) and non-consensus (small nt) are underlined. Previously predicted promoter elements (-10_1 & -35_1 and -10_2 & -35_2) for *caf1M* [18] and BPROM predicted promoter elements, -10_3 & -35_3 are underlined. Identified repeats are labelled as R1-R3, predicted *caf1R* autoregulation (red arrows) and R3′ and R4′ for *cafMA1* (black arrows), small nt- variants. (**B**) Alignment of the identified repeats with conserved (yellow) and variant nt (green). Absolutely conserved nt among these repeats are indicated as *. The consensus motif for the MarA/Rob/SoxS A/X family [22] is- R: A/G, Y: C/T, W: A/T and N: A/T/G/C. The predicted binding sites of R4′ sequence interacting with proposed helix-3 (BS1) and helix-6 (BS2) of the Caf1R is bold underlined (identified based on *mar*-MarA co-crystal structure [25]. (**C**) Schematic of the *caf1R-caf1M* transcriptional *lacZ* fusions. The largest construct has the entire intergenic regions plus +192 and +158 flanking sequence from *caf1R* and *caf1M*, respectively. Location of truncations within the intergenic region are numbered with respect to ATG of *caf1M* and are indicated by a blue arrow in (**A**). The β-galactosidase activity of the fusions, with and without plasmid complementation of Caf1R and fold activation in presence of Caf1R is indicated. Standard error of mean (± SEM) is from three biological replicates. (**D**) EMSA, *in-vitro* binding of MBP-Caf1R*/R_N_ to the oligonucleotide [47 bp] encompassing R4′ (blue underlined sequence in (**A**). MBP-Caf1R*, MBP-Caf1R_N_ and MBP, cell lysates from *E. coli* K12 2508 expressing the relevant recombinant protein. Ctrl- reaction lacking cell lysate. UB- unbound R4′ DNA and B- R4′ DNA complex with MBP-Caf1R*/R_N_.

The F1 encoding *caf* locus is located on the *Y. pestis* specific virulence plasmid, pFra1, also called pMT1 [58] and codes for four genes which are divergently transcribed (Figure 5A). Three potential promoters were predicted by bioinformatic analysis of the intergenic region between *caf1R-caf1M* when the locus was initially sequenced [18], one for *caf1R* and the other two for *caf1M.* No such promoter had been predicted for the intergenic region of *caf1A-caf1*, suggesting the intergenic region of *caf1R-caf1M* is the main regulatory region of the *caf* genes cluster controlling the expression of *caf1R* in one direction and *cafMA1* operon in the opposite direction [59]. The *caf1R-caf1M* intergenic region was re-analysed for potential promoters. A single promoter was predicted by BPROM with close proximity to *caf1M*, -104 bp upstream of *caf1M* start codon, having -10 (TATAAA) and -35 (TTCTCA) elements with 18 nt spacer (Figure 5A). This prediction indicates a better score for the -10 element, which is in good agreement with σ^70^ bacterial promoters where one element shows a higher consensus conservation than the other with 17 nt spacer [60]. Following visual analysis, three additional potential promoters were predicted within the *caf1R-caf1M* intergenic region, two upstream of *caf1R* [59] and another for *caf1M*, labelled P_M_ with consensus motifs, -10 (TAAAAT) and -35 element (TAAACT) and 17 nt spacer (Figure 5A). The sequence composition of these three additional putative promoters also indicates a stronger -10 element with a spacer of 17–18 nt from the corresponding -35 element [59].

### 2.8. Identification of Potential Caf1R Binding Motifs

Five repeat motifs of 15 nt long, labelled R1-R3 and R3′-R4′ were identified within the *caf1R-caf1M* intergenic region (Figure 5A). The R1-R3 repeats were identified at 56–70 bp (R1), 89–103 bp (R2) and 122–136 bp, (R3) upstream from the ATG start codon of *caf1R*, with 18 nt spacer between each. Repeat motifs R3′ and R4′ run in the opposite orientation at 163–177 bp (R4′) and 183–197 bp (R3′) upstream from the ATG start codon of *caf1M*, with a 5 nt spacer. There are 14 nt between R3 (for *caf1R* motif) and R4′ (for *cafMA1*). Importantly, R4′ overlaps the -35 consensus sequence of the predicted P_M_ promoter for *cafMA1*, a typical feature of Class II regulatory activators [63,64]. Following alignment of these five repeat motifs (Figure 5B) and comparison with DNA motif from P*mar*-MarA co-crystal structure [25] and consensus DNA binding motif of the MarA/Rob/SoxS A/X family regulators [22], a potential Caf1R-binding consensus was determined, indicating a strong similarity with the MarA/Rob/SoxS binding consensus (Figure 5B).

### 2.9. In Vivo Validation of the Identified Caf Promoters and Caf1R-Binding 

To validate the predicted promoters and potential Caf1R binding repeat motifs, the five progressively shorter *lacZ* reporter fusions upstream of *cafMA1* shown in (Figure 5C) were constructed in the *lacZ* transcriptional reporter plasmid pRS550 [65]. These fusions were designed to progressively remove the predicted Caf1R binding sites and promoter elements highlighted on the intergenic DNA sequence shown in Figure 5A. The β-galactosidase activity was monitored, as described in methods, with and without plasmid complementation of Caf1R (from pACYC-R). There was no evidence of promoter activity with or without Caf1R when the *caf* DNA sequence includes only residues from −141 to +158 (where +1 is the start codon of *caf1M*). Thus, it can be concluded that none of the predicted promoters within this region are functional under the conditions tested. Results with plasmid pDKG*caf1M′*_−169+158_-*lacZ* recorded promoter activity, but no Caf1R activation. In contrast, inclusion of DNA encompassing the predicted Caf1R binding site, R4′ (in pDKG*caf1M′*_−184+158_-*lacZ*) led to a 10.53-fold activation in the β-galactosidase activity. Thus, the results with these latter two plasmids (pDKG*caf1M′*_−169+158_-*lacZ* and pDKG*caf1M′*_−184+158_-*lacZ*) identify P_M_ as the Caf1R-dependent promoter for activation of transcription from the *cafMA1* operon. This also identifies the repeat sequence R4′ as the Caf1R binding site and a suitable target for EMSA studies. In the promoter fusion assays, there was a further small increase in activation, to 12.8-fold when R3′ was also included (Figure 5C). The significance of this requires more detailed analysis in relation to binding and activation by Caf1R. 

### 2.10. In-Vitro Binding of MBP-Caf1R* to the CafR4′ Motif

To verify the activity of the MBP-Caf1R* fusion protein, an in vitro DNA-protein interaction assay (EMSA) was performed using a synthetic oligonucleotide encompassing the identified R4′ motif as target (Figure 5A; underlined in blue). EMSA with cell lysates from *E. coli* K12 ER2508 expressing MBP-Caf1R* showed a prominent shift of R4′ *caf* DNA motif confirming correct folding and DNA-binding functionality of Caf1R when fused to MBP. A plasmid encoding MBP-Caf1R_N_ (containing only N-terminal DNA binding domain of Caf1R) had also been constructed (Figure S9). Therefore, this was also tested post-expression in *E. coli* K12 ER2508 expressing MBP-Caf1R_N_. This MBP fusion with Caf1R-DBD domain alone induced the same prominent shift of R4′ *caf* DNA motif (Figure 5D). No shift was induced by MBP alone or in the negative control without cell lysate. 

Conclusively, MBP-Caf1R* overproduced from synthetic codon-optimised *caf1R* gene, was shown to be suitable for use in DNA binding studies and can presumably also be used for other Caf1R-related functional assays to get a deeper insight into the Caf1R-regulated pathophysiology of *Y. pestis*. Moreover, this in vitro assay combined with the promoter fusion study has demonstrated that native Caf1R binds strongly at the -35 element of identified P_M_ promoter, encompassing Caf1R-binding R4′ motif, to control transcription of the *cafMA1* operon. 

## 3. Discussion 

*Yersinia pestis* F1-capsule regulator (activator), Caf1R, is a member of the widely distributed A/X family of the bacterial transcription factors/regulators. Regulators belonging to this family are found in >80% of non-redundant prokaryote genomes and are involved in regulating genes essential in central metabolism, stress-response and virulence [19]. A great number of proteins in this family are notoriously difficult to purify in their native functional state. This is reflected in the fact that to date only a small number of these proteins have been functionally characterised, to be precise, 126 out of 15, 935 as analysed by Cortes-Avalos et al. [19]. This problematic property (prone to aggregate) could in part be due to the differences in the pI of their two domains- a signatory DNA binding domain (DBD) and a variable sensing or oligomerisation domain [19,20]. However, expressing one domain in isolation resulted in soluble protein for only some member proteins [52,53,54] and this is not the case for a large proportion of A/X family proteins. The high number of Cys residues in the sensing/oligomerisation domain of Caf1R likely also contributes to aggregation problems.

Despite problems associated with A/X family proteins, a handful of proteins of this family have been successfully over-expressed, purified and subjected to in-detail structural studies, including the putative-structural homologs of Caf1R: MarA, Rob and XylR proteins of *E. coli* [24,30,66]. Here, Caf1R expression was monitored from both the native and synthetic codon-optimised genes of Caf1R. Both native and synthetic genes were sub-cloned in three frequently used expression plasmids for the A/X family regulators. Two contain N-terminal small His_6_-tag, pBADHisA and pET28a^+^ under control of the P_BAD_ and T7 promoter, respectively. The third plasmid, pMALc2x encodes an N-terminal solubility enhancer tag (MBP) under control of the IPTG inducible P*tac* promoter.

Native expression of His-Caf1R from the pBADHisA plasmid resulted in very low recovery of soluble protein, detected only by immunoblot. The in vivo, trans-complementation studies, demonstrated that pBADhCaf1R expressed His-Caf1R functioned efficiently to activate expression of the *cafMA1* operon, and lead to production of high levels of surface F1 fibres. Thus, the N-terminal His-tag does not interfere with function of the N-terminal DBD domain of Caf1R. To boost the level of expression of His-Caf1R, both native and synthetic codon-optimised *caf1R* were cloned into the over expression vector pET28a^+^, as pET vectors had been successfully used in the over expression of putative-structural homologs of Caf1R, MarA [67], Rob [24] and XylR [30]. Surprisingly, the level of native His-Caf1R from pET28a^+^ plasmid was not substantially higher than that from pBADHisA plasmid. However, the level of expression was dramatically improved using a codon-optimised caf1R*. When expressed from the pET28a^+^ (His-Caf1R*) high levels of production required supplementation of cultures with glucose throughout growth. Relief from IPTG-mediated substrate toxicity [57], by inclusion of glucose, can be explained by catabolite repression of the *lacUV5* promoter controlling T7 polymerase expression in *E. coli* BL21 and hence more controlled expression of encoded recombinant proteins. 

Expression of His-Caf1R* from the pET28a vector resulted mainly in recovery of protein in inclusion bodies, including following expression at lower temperatures. The abundant His-Caf1R* from excessive inclusion bodies (from pET28a^+^) could also represent a suitable source of potentially functional Caf1R if used in solubilisation and refolding trials. Refolding of MarA from inclusion bodies led to successful resolution of the X-ray crystal structure of MarA-DNA complex [25]. 

The MBP-Caf1R* fusion was the most successful construct for production of soluble Caf1R. MBP has been shown to enhance the solubility of several A/X regulators and their subsequent characterisation without removing the MBP-tag. Examples include SoxS [32], Rns [68] and PchR [34]. The MBP-Caf1R from the native *caf1R* was found to be susceptible to degradation when expressed in BL21(DE3). On the other hand, *E. coli* K12 strain ER2508 which is deficient in major ATP-dependent proteases worked as an excellent host strain for the over-expression of MBP-Caf1R/R*. Soluble MBP-Caf1R* represented the major protein in cell supernatants from *E. coli* ER2508/pMBP-Caf1R*. This contrasted with the requirement to use immunoblotting to identify soluble His-Caf1R/R*. Importantly, MBP-Caf1R* has already been used here to confirm identity of the Caf1R binding site, R4′ *caf* DNA motif, and P_M_ promoter that drives transcription of the *cafMA1* operon.

**Figure 6:**
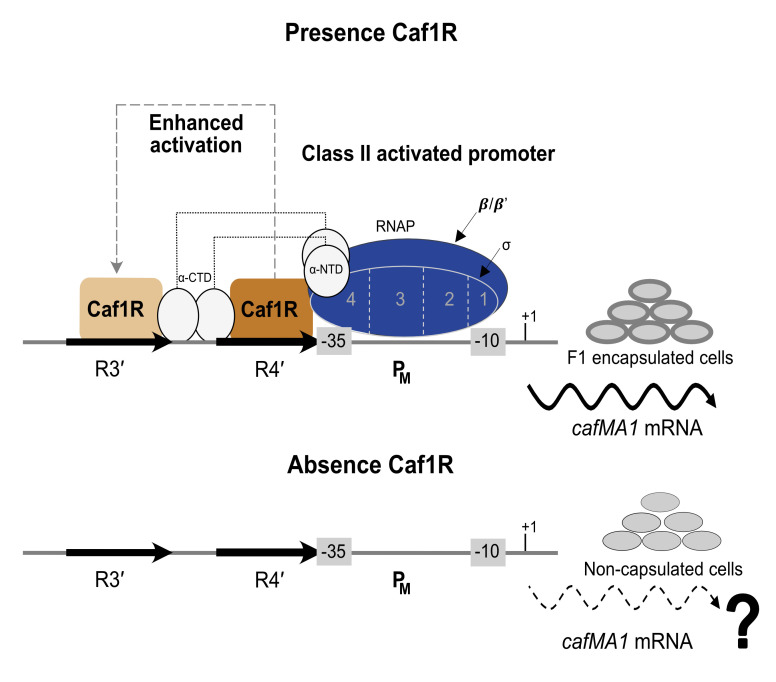
Model of Caf1R-dependent transcription activation at P_M_ promoter of the *caf* locus. Based on published evidence of RNA polymerase (RNAP)-regulator interactions, it is proposed that Caf1R either initially interacts with RNAP and then binds at the R4′ *caf* DNA motif or first binds to the R4′ *caf* DNA motif and then interacts with RNAP to enhance RNAP-Caf1R interaction at the P_M_ promoter for initiating transcription of the *cafMA1* operon. In addition, to this Class II activation on binding at P_M_, the diagram also depicts the possibility of further activation of transcription, in a Class I manner, via binding of Caf1R at the R3′ *caf* DNA repeat motif. Expression of *cafMA1* leads to abundant F1 level on the cell surface, giving the appearance of encapsulated cells. In the absence of Caf1R there is little or no expression of *cafMA1*.

Based on these results, a model depicting Caf1R-mediated transcription activation of the *cafMA1* operon at the P_M_ promoter leading to production of F1-encapsulated cells at 37 °C is presented (Figure 6). The promoter fusion studies combined with EMSA analysis using MBP-Caf1R* have identified the functional promoter, P_M_ for expression of the *cafMA1* operon. This is preceded by the R4′ binding site which also overlaps the promoter -35 region. This organisation of the binding site is characteristic of Class II activator regulators and the diagram includes the characteristic interaction of these activators with domain 4 of the RNA polymerase sigma subunit [22,63,64,69,70]. Caf1R regulator may either initially interact with RNA polymerase and then bind to R4′ *caf* DNA motif. Alternatively, it could first bind to R4′ *caf* DNA motif and then interact with RNA polymerase to enhance its binding and kinetics of activation at the P_M_ promoter. Presence of the R3′ repeat further upstream from the confirmed R4′ Caf1R binding site was not essential for a high level of activation of transcription. However, there was a small enhancement in activation of transcription in the promoter fusion study, with inclusion of R3′ repeat sequence. Hence, the possibility that Caf1R may also function as a Class I activator could be explored and has been outlined in Figure 6. We have also studied the auto-regulation of *caf1R* [59] and this represents an additional fascinating area for future focus which will contribute to the overall understanding of the A/X family of bacterial regulators. The availability of functional tagged and purified fusion constructs of Caf1R will be valuable tools to further explore the molecular complexity of interaction of the Caf1R regulator with this important locus of the pathogen *Yersinia pestis.*

## 4. Materials and Methods

### 4.1. Bacterial Strains, Plasmids, DNA Oligos, and Culture Conditions

DNA oligos/primers (purchased from Eurofins Genomics, Ebersberg, Germany), plasmids and *E. coli* strains used in this study are listed in Tables S1–S3. Bacteria were routinely cultured in Luria Bertani (LB) broth or agar at required temperatures with agitation. Where required, media were supplemented with ampicillin [100 μg/mL], chloramphenicol (10–34 μg/mL) or kanamycin (30 μg/mL). 

### 4.2. Plasmid Design 

Unless otherwise specified, the plasmids were designed as per InFusion molecular cloning (Clontech-Takara, UK). Briefly, native or synthetic-codon optimised *caf1R* was PCR-amplified using respective InFusion primers (Table S1). PCR-product was gel purified and InFused in the corresponding linearised plasmid (linearisation by restriction digestion). InFusion mixture was transformed into competent Stellar^TM^ cells (Table S3) and transformants were screened by colony PCR using the same InFusion primer pairs that were used for the cloning. Plasmid DNA was routinely prepared using Qiagen mini-prep as per manufacturer’s instructions. Intact fusion of *caf1R* without any mutation was confirmed by sequence analysis. Sequencing was performed either from Source Bioscience (Oxford, UK) or Eurofin Genomic (Ebersberg, Germany). A schematic of designed plasmids is provided in the Supplementary.

### 4.3. Optimisation of Heterologous Expression of Caf1R

Expression of recombinant Caf1R was monitored from its native and synthetic codon-optimised gene. Three established expression plasmids including pBADHisA (Novagen^TM^), pET28a+ (Novagen^TM^) and pMALc2x (New England Biolabs, Hitchin, UK), which differ in their expression capability for the recombinant proteins, were utilised. Expression level of recombinant Caf1R from these plasmids was examined in *E. coli* host strains library, equipped with unique features (Table S3), as indicated in Results.

### 4.4. Sampling of Recombinant Caf1R

LB broth (10 mL) supplemented with appropriate antibiotic (Amp. 100 µg/mL and Cm. 10 µg/mL, as required) was inoculated with a single isolated fresh colony of *E. coli* strain expressing desired plasmid. Cultures were grown at 37 °C with shaking (225 rpm) for 16–17 h. Next day, cells from 1–5 mL culture were centrifuged (13,000 rpm, 5 min) and washed with LB (13,000 rpm, 5 min). Washed cells were then re-suspended in the original volume of LB and inoculated (1/100) in selective LB, containing the appropriate antibiotic and 1% glucose (for plasmids containing codon-optimised caf1R*). Small-scale (10 mL) cultures were grown in 20 mL glass tubes while for large-scale (500 mL), 2L Erlenmeyer flasks were used. Subcultured cells were grown at 37 °C on a rotary shaker (225 rpm) until ~0.5 optical density (OD) at 600 nm. At this point, cells were induced with either different concentrations of IPTG (for pET28a+ and pMALc2x-based constructs) or 0.02% L-arabinose (for pBADHisA-based constructs). Following induction, cultures were either grown at 37 °C or moved to lower temperatures, as indicated. For an initial expression analysis, an equal number of induced and non-induced cells (1 OD unit at Abs 600 nm) were harvested by centrifugation (13,000 rpm–5 min) and lysed by sonication (pulse-10, amplitude-50, time-5 min) on ice in desired buffer (100 μL), supplemented with 1× complete proteases inhibitor cocktails (Sigma Aldrich, Gillingham, UK). For large-scale procedures, cells were recovered at 4000 rpm/20 min at 4 °C and re-suspended directly in the lysis buffer of choice (100 μL/OD). For Caf1R-expression constructs, based on pBADHisA and pET28a+ plasmids backbone, EDTA-free proteases inhibitor was added during cell lysis whereas for pMALc2x-based constructs, EDTA-plus proteases inhibitor was used throughout. 

### 4.5. Preparation of Cell Lysate and Isolation of Soluble Recombinant Caf1R 

Cells were routinely lysed by sonication using Sonic Vibra^TM^ Sonicator (SONICS^®^). Samples were always kept on ice and sonicated with correct sized probe until complete cell-lysis. Depending on batch volume, cells were lysed for 5–15 min, with 10 s pulse at 50% amplitude. Cells lysed by Bug buster master mix (Novagen) did not require sonication. To eliminate residual unlysed cells, lysate was clarified by centrifugation at 4000 rpm/20 min at 4 °C. Subsequently, cell debris (membranes) and inclusion bodies were removed by centrifugation at 20,000 rpm/20 min at 4 °C. To isolate soluble protein, lysate supernatant was further clarified by ultracentrifugation at 50,000 rpm (134, 877× *g*) for 60 min at 4 °C using TLA-100.3 rotor. The supernatant fraction was assigned as ‘S’. Pelleted cells from 20,000 rpm and 50,000 rpm were combined and mixed in the same volume of buffer that was used for cell lysis. This insoluble protein suspension was assigned as ‘P’. Both S and P samples were mixed with an appropriate volume of 4× SDS-PAGE sample buffer (10% glycerol, 62.5 mM Tris-HCl pH 6.8, 8% SDS, 0.1 mg/mL bromophenol blue) supplemented with DTT [200 mM] to give a final concentration of ×1 and heat-denatured (95 °C−10 min) for subsequent analysis by SDS-PAGE and Western immunoblotting.

### 4.6. Purification of Soluble MBP-Caf1R* by Amylose Affinity Chromatography

Cell lysate was prepared from 500 mL culture of *E. coli* K12 ER2508/pMALCaf1R*, induced with 0.30 mM IPTG at 25 °C for 5 h. Induced cells were recovered by centrifugation and mixed in 40 mL of MBPTrapHP binding buffer (20 mM Tris-HCl, 200 mM NaCl, 1 mM EDTA and 10 mM β-Mercaptoethanol; pH 7.45) supplemented with 1× protease inhibitor (Thermo Scientific, Pierce^TM^, Loughborough, UK) followed by sonication and ultracentrifugation (134,877× *g* for 60 min at 4 °C). Using an ÄKTA purifier (GE Healthcare, Chalfont Saint Giles, UK), supernatant (35 mL) was applied to an equilibrated 1 mL MBPTrapHP column (GE Healthcare, Chalfont Saint Giles, UK), equilibrated in binding buffer at a rate of 0.5 mL/min. Following column wash, bound MBP-Caf1R* was eluted with 12 column volume of the column buffer over a linear gradient of 10 mM maltose, collecting 12 fractions (1 mL each). 

### 4.7. SDS-Polyacrylamide Gel Electrophoresis (SDS-PAGE) and Western Immunoblotting

SDS-PAGE was performed using the classical Laemmli system and 14% acrylamide gels. Western immunoblotting was routinely used to validate the expression level of recombinant Caf1R. Nitrocellulose membrane (Amersham Biosciences, Little Chalfont, UK) was used throughout. The His_6_-tagged variants were identified with antiHis-HRP monoclonal antibody (Roche, Basel, Switzerland) [1:10,000 dilutions]. Identity of MBP-tagged variants were confirmed using polyclonal anti-MBP (New England Biolabs, Hitchin, UK) as primary and anti-Rabbit-HRP (Amersham Biosciences, Little Chalfont, UK) as secondary antibodies-both in 1:10,000 dilutions. Expression level of both His_6_- or MBP-tagged Caf1R variants was verified and quantified in comparison with cells expressing empty plasmid (negative control). Chemiluminescence substrate, ChemiFast (Syngene, Cambridge, UK) was used for detection based on the manufacturer’s instructions. 

### 4.8. Protein Quantification

Nanodrop ND-1000 spectrophotometer (Thermo Scientific, Loughborough, UK) was used to estimate the protein concentration in the purified samples of recombinant Caf1R. The inbuilt parameter, 1A/cm = 1 mg/mL was considered to calculate recombinant Caf1R concentration which was further reassessed by classical Bradford assay. 

### 4.9. Bioinformatic Analysis

Unless otherwise specified the following bioinformatic tools were used. The DNAdynamo (https://www.bluetractorsoftware.com/, accessed on 18 June 2014) was routinely used to design primers, plasmids and analysis of sequencing results. Raw sequencing data- chromatogram peaks were regularly analysed by FinchTV (https://digitalworldbiology.com/FinchTV, accessed on 18 June 2014). The REPFIND tool (http://cagt.bu.edu/page/REPFIND_submit, accessed on 18 June 2014) was used for the initial identification and analysis of DNA repeat motifs and the BPROM [71] was used to localise potential Caf1R-regulated promoters. 

### 4.10. Statistical Analysis

ImageJ [72] was used to quantify expression level of full-length recombinant Caf1R in comparison with negative control, i.e., cell expressing corresponding empty plasmid. The numbered values from peak area of the full-length recombinant Caf1R band or corresponding area from the negative control were inferred either in percentage or relative level. GraphPad Prism-7 (San Diego, CA, USA) was used to generate the graphs. 

### 4.11. Functional Analysis of the Caf1R

#### 4.11.1. Functional Confirmation of His-Caf1R by Trans-Complementation of pACYC-MA1 and F1 Production

The *E. coli* Top10 cells co-transformed with pBADhCaf1R + pACYC-MA1 were cultured in 10 mL LB (plus Amp and Cm) at 37 °C to mid-logarithmic phase (OD600 nm 0.5) with shaking at 200 rpm, followed by 4 h induction with 0.02% L-arabinose. Surface F1 was extracted from induced cells as previously described [73]. Briefly, cell pellets from 3 OD_600_ units cell culture were recovered at 13,000 rpm for 20 min suspended in 100 μL of PBS, pH 7, and heated at 56 °C for 60 min to extract F1 fibres. Following removal of cells by centrifugation (13,000 rpm for 10 min), supernatants containing F1 polymer were prepared for analysis by SDS-PAGE (16% acrylamide) either without heating to detect F1 polymer or by heating at 100 °C for 10 min in SDS-PAGE sample buffer to denature polymer and identify Caf1 subunit.

#### 4.11.2. Identification of Potential Caf1R Binding Site(s) and Caf1R-Regulated Promoter(s)

The key regulatory region of the *caf* genes cluster, *caf1R-caf1M* intergenic region [327 bp] with several hundred flanking bases was examined by REPFIND with default parameters. Identified repeats (3–7 nt) were further analysed visually on both strands to increase the length of each repeat including mismatches. The direction of each repeat was assigned with respect to closeness to the preceding gene. Further, the same DNA fragment was analysed for prediction and identification of the *caf* promoters using BPROM [71]. Sequence motifs with highest similarity with consensus of -10 (TATAAT) and -35 (TTGACA) elements, having 16–22 nt spacer were also inspected visually. 

#### 4.11.3. Lysed Cell β-Galactosidase Assay

The β-galactosidase activity was assayed using whole cell lysates essentially as described [74] to monitor activity of promoter-*lacZ* reporter fusions, with and without plasmid complementation of Caf1R (from pACYC-R). The *E. coli* Top10 cells [0.5 unit at OD600 nm] from an ON culture of cells freshly transformed with the relevant-*lacZ* reporter fusion ± pACYC-R were inoculated into 50 mL LB containing Amp (100 µg/mL) and Cm (10 µg/mL) and incubated at 37 °C with shaking at 225 rpm. OD_600_ was monitored and 0.5 OD unit of culture harvested every 2 h for 10 h. Bacteria were recovered at 13,000 rpm for 5 min and pellets immediately frozen (−20 °C). To measure β-galactosidase activity, frozen pelleted cells were resuspended in 100 μL of Bugbuster master mix (Merck Millipore Novagen^TM^, Thermo Fisher Scientific, Loughborough, UK) solution and incubated at 37 °C for 20 min with gentle shaking. The cell lysate was centrifuged (13,000 rpm–5 min) and the supernatant stored on ice and used immediately to measure β-galactosidase activity. Assays were performed from triplicate cultures from 3 different transformants for each construct, in 96-well flat bottomed transparent microtitre plates with 4 μL cell lysate samples and 196 μL Z-buffer (1.0 mM MgSO_4_, 10 mM KCl, 60 mM Na_2_HPO_4_, 40 mM NaH_2_PO_4_, 14.9 mM ONPG, 24.9 mM DTT, 8.85% PBS; pH 7.5) to start the reaction. Change in absorbance at 420 nm was recorded kinetically using a SpectraMax Plus Microplate reader (Molecular Devices Corps, San Jose, CA, USA) for an hour at 30°C and the V_max_ calculated. The β-galactosidase activity was defined as the V_max_ /OD_600_ cell culture. 

#### 4.11.4. In Vitro Binding of Recombinant Caf1R to the Identified R4′ Repeat 

A LightShift^®^ Chemiluminescent EMSA kit (Thermo Fisher Scientific, Pierce^TM^, Loughborough, UK) was used to monitor in vitro activity of recombinant MBP-Caf1R*. The 3′ Biotin-TEG (Eurofin, Ebersberg, Germany) labelled R4′-motif (47 bp) complementary strands (100 μM each) were annealed in a reaction buffer (10 mM Tris-HCl, pH 7.5, 100 mM NaCl, 1 mM EDTA) at T_m_ + 10 °C for 10 min and then gradually cooled to room temperature for 1 h. Cell-lysates for EMSA were prepared by growing recombinant cells in 10 mL LB broth (containing 100 μg/mL ampicillin) at 37 °C overnight, with shaking at 225 rpm. The next day, cells were subcultured (1/100) in 10 mL LB and grown at 37 °C, with shaking until 0.5 OD600 nm. Cultures were induced with 0.3 mM IPTG for 2.5 h. Induced cells were harvested (1.0 OD unit) by centrifugation (13,000 rpm–5 min), resuspended in 500 μL EMSA lysis buffer (10 mM Tris-HCl, pH 7.5, 1 mM EDTA, 100 mM KCl, 5 mM MgCl_2_, 1 mM DTT, 10% Glycerol and 1× protease inhibitor) and lysed by sonication (on ice) for 3 min with 25 s pulse at 50% amplitude. Cell lysate was clarified by centrifugation (21,000 rpm/15 min/4 °C), and the supernatant fractions used in EMSA reactions. A 20 μL EMSA reaction was set up at room temperature by mixing 6 μL cell lysate with 10 fmol Biotin labelled R4′-motif and 1 μL of 1 μg Poly dI.dC in the EMSA binding buffer (10 mM Tris, 50 mM KCl, 1 mM DTT; pH 7.5). The reaction mix was mixed gently and incubated at room temperature for 20 min. To each reaction 5 μL of 5× EMSA loading buffer (Thermo Fisher Scientific, Pierce^TM^, Loughborough, UK) was added and the whole reaction mixture (25 μL) was resolved on Bolt^TM^ Mini DNA-retardation gels, containing 6% acrylamide (Thermo Fisher Scientific, Life technologies, Loughborough, UK). Electrophoresis was carried out in 0.5× TBE buffer (44.57 mM Tris-base, 44.47 mM Boric acid, 0.025 mM EDTA; pH 8.0) at 100 volts for 90 min at 4 °C. Resolved EMSA reactions were electro-transferred on Nylon Hybond XL membrane (GE Healthcare, Chalfont Saint Giles, UK) at 30 volts for one hour at 4 °C using 0.5× TBE. Membrane was UV cross-linked for 5 min using T-20 UVP Dual-intensity trans-illuminator and immunoblotted with streptavidin-HRP conjugate as per manufacturer directions (Thermo Fisher Scientific, Pierce^TM^, Loughborough, UK). Membrane was pictured using G-box (Syngene, Cambridge, UK) with auto-time exposure. 

## Data Availability

Not applicable.

## Supplementary Materials

The following are available online at https://www.mdpi.com/article/10.3390/ijms22189805/s1.
