# Supplementary material for: Optimised Heterologous Expression and Functional Analysis of the Yersinia pestis F1-Capsular Antigen Regulator Caf1R
Source: Int J Mol Sci. 2021 Sep 10;22(18):9805. doi: 10.3390/ijms22189805 (PMC8470410; doi:10.3390/ijms22189805)
Supplement: Supplementary file 1 [file ijms-22-09805-s001.zip › ijms-1345460 Supplementary.pdf]

SUPPLEMENTARY INFORMATION

Optimised Heterologous Expression and Functional Analysis of the *Yersinia pestis* F1-Capsular Antigen Regulator Caf1R

Dharmender K. Gahlot[1,2,3*], Gyles Ifill[1,4] and Sheila MacIntyre[1*]

[1]School of Biological Sciences, University of Reading, RG6 6UR Reading, United Kingdom
[2]Department of Molecular Biology, Umeå University, 901 87 Umeå, Sweden
[3]Umeå Centre for Microbial Research (UCMR), Umeå University, 901 87 Umeå, Sweden
[4]Department of Microbiology and Immunology, University of British Columbia, V6T 1Z3, Vancouver, British Columbia, Canada

***Correspondence:** dharmender.kumar@umu.se (D.K.G); s.macintyre@reading.ac.uk (S.M.)

Tables

Table S1. DNA oligos/primers used in this study.

Table S2. Plasmids used in this study.

Table S3. *E. coli* strains used in this study.

Figures

Figure S1. The pBADhCaf1R plasmid construct.

Figure S2. The pEThCaf1R plasmid construct.

Figure S3. Expression of His-Caf1R (native *caf1R*) from *E. coli* BL21(DE3).

Figure S4. Sequence alignment of native Caf1R with codon-optimised Caf1R*.

Figure S5. The pEThCaf1R* plasmid construct.

Figure S6. The pMALCaf1R plasmid construct.

Figure S7. The pMALCaf1R* plasmid construct.

Figure S8. Expression of MBP-Caf1R (native *caf1R*) from *E. coli* BL21(DE3) and K12 ER2508.

Figure S9. The pMALCaf1R$_N$ plasmid construct.

Figure S10. The pACYC-R and pACYC-MA1 plasmid constructs.

Table S1. DNA oligos/primers used in this study.

Plasmid	Primer	Sequence (5′-3′)	GC (%)	Tm (°C)
		**InFusion primers**[1]		
**pEThCaf1R**	pETCaf1Rf	**CAGCCATATGGCTAGCAAG**GCTAGCTGGGGATCC**GAGCTCATTTG** ***Nhe*I**	57.7	68.0
	pETCaf1Rr	**GACGGAGCTCGAATTCTT**CGAATTCCCATATGGTACCAGC ***Eco*RI**	50.0	60.3
**pMALCaf1R**	pMALCaf1Rf	**AAGGATTTCAGAATTC**GTTGAATTCATGCTAAAACAGATG ***Eco*RI**	33.3	55.9
	pMALCaf1Rr	**CGACTCTAGAGGATCC**TATGGATCCAGCTGCAGATC ***Bam*HI**	50.0	57.3
**pMALCaf1R***	pMALCaf1R_GSf	**AAGGATTTCAGAATTC**ATGCTGAAACAAATGACCGTGAAC ***Eco*RI**	41.7	59.3
	pMALCaf1R_GSr	**CGACTCTAGAGGATCC**TTATGATTTCGGCAGACCCAGCGT ***Bam*HI**	50.0	62.7
**pMALCaf1R$_N$**	pMALCaf1RNifF	**AAGGATTTCAGAATTC**TCCGAGCTCATTTGGGTTATATTC	41.7	59.3
	pMALCaf1RNinfR	**CGACTCTAGAGGATCC**TTAAAAGGACCAAAAAGGGATCATC TTA–**Stop codon**	36.0	58.1
**pDKG*caf1M′*$_{-197+158}$-*lacZ***	pRS550(13-16)F; Delta1for	**CAGGAATTGGGGATC**TGCGCAACAAGCAAGTGGAGTG	50.0	62.7
	pRS550(13-16)R	**GTCCGGATCGGAATT**CTAACGGGTATATGATCCTACTCTC	44.0	61.3
**pDKG*caf1M′*$_{-184+158}$-*lacZ***	Delta 15F	**CAGGAATTGGGGATC**AGTGGAGTGCGC	66.7	40.0
	Delta 550R	**GTCGGATCGGAATT**CTAACGGGTATATGATCC	44.4	51.4
**pDKG*caf1M′*$_{-169+158}$-*lacZ***	Delta 14F	**CAGGAATTGGGGATC**AAGCTAAACTTTGTGTG	35.3	45.5
	Delta 550R	**GTCGGATCGGAATT**CTAACGGGTATATGATCC	44.4	51.4
**pDKG*caf1M′*$_{-141+158}$-*lacZ***	Delta4for	**CAGGAATTGGGGATC**AAAATTGTTCTCAGTGAGGCTG	40.9	56.5
	pRS550(13-16)R	**GTCCGGATCGGAATT**CTAACGGGTATATGATCCTACTCT	44.0	61.3
**pDKG*caf1M′*$_{-102+158}$-*lacZ***	pRS550(13-17)F or Delta2	**CAGGAATTGGGGATC**TCCCCTTCATTTGTTACCCACC	44.4	51.4
	pRS550(13-16)R	**GTCCGGATCGGAATT**CTAACGGGTATATGATCCTACTCTC	44.0	61.3
**pACYC-R**	pACYC*caf*1Rf	GCCTAACCCTC**GAGCTC**G ***Sac*I**	66.7	60.5
	pACYC*caf*1Rr	GCTT**GAGCTC**CTTACGGAATG ***Sac*I**	52.4	59.8

**pACYC-MA1**	Inv*caf*1Rfor	AC**CCATGG**CAAAATAATAGCATTCTAGATAGTG ***Nco*I**	36.4	64.5
	Inv*caf*1Rrev	TC**CCATGG**CAGGAGTCGCATAAGG ***Nco*I**	58.3	66.1
		**Sequencing primers to confirm**		
**Recombinant *caf1R/R** in pET28a+ plasmid**	T7F	TAATACGACTCACTATAGGG	40.0	48.0
	T7R	GCTAGTTATTGCTCAGCGG	53.0	51.0
**Recombinant *caf1R/R** in pMALc2x plasmid**	*malE* for	GGTCGTCAGACTGTCGATGAAGCC	58.0	61.0
	M13F	TGTAAAACGACGGCCAGT	50.0	48.0
***caf1R* and *caf1R-caf1M* intergenic region**	ACYCDuetUp1	GGATCTCGACGCTCTCCCT	63.0	55.0
	DuetDown1	GATTATGCGGCCGTGTACAA	50.0	52.0
***caf1A-caf1* intergenic region**	F1for 11	GTGGATCCCTGGCATTGTCGGAGATAATAGCG	53.0	62.7
	F1rev 12	CGGAATTCGCAGTGGTGCTTGCAGTTAAATC	54.0	60.6
***lacZ*-fusion**	F1_6	CCTTCTGCTGAATCCTGAATAC	45.0	53.0
	M13F	TGTAAAACGACGGCCAGT	50.0	48.0

[1]DNA bases from vector ends are indicated in bold capital text and T_m is shown only from gene specific part, as per InFusion cloning (Clontech-Takara).

Table S2. Plasmids used in this study.

Plasmid	Description	Source
**pBADHisA**	Low copy number, pBR322-derived expression plasmid (4.1 kb) contains N-terminal His$_6$-tag and L-arabinose inducible P$_{BAD}$ promoter. Amp^R	Invitrogen, UK
**pBADhCaf1R**	pBADHisA based plasmid contains native *cpxR* in between *Sac*I and *Bgl*II restriction sites.	Alma Lopez-Tolman; SM lab stock
**pET28a$^+$**	High copy number (~ 40), pBR322-derived expression plasmid (5.369 kb), contains N-terminal His$_6$-tag and T7 promoter, induction by IPTG. Kan^R	Novagen, UK
**pEThCaf1R**	pET28a$^+$ based plasmid, contains N-terminally His$_6$-tagged native *caf1R*.	This study
**pMAL-c2x**	Medium copy number (~20), pMB1-derived expression plasmid vector (6.646 kb), contains N-terminal maltose-binding protein (MBP)-tag and P*tac* promoter. Specific for over-expression of cytoplasmic protein, induction by IPTG. Amp^R	NEB, UK
**pMALCaf1R**	pMAL-c2x based plasmid, contains N-terminally MBP-tagged native *caf1R.*	This study
**pEThCaf1R***	pET28a$^+$ based plasmid, contains N-terminally His$_6$-tagged synthetic codon-optimised *caf1R*, cloned in between *Eco*RI and *Nhe*I sites.	GenScript, USA
**pMALCaf1R***	pMAL-c2x based plasmid, contains N-terminally MBP-tagged synthetic codon-optimised full-length *caf1R.*	This study
**pACYC-F1**	Contains *Yersinia pestis* EV76 *caf* locus (5.128 kb) with 188 and 175 bp flanking ends (relative to *caf1R* and *caf1* stop codon), cloned in between *Nco*I and *Sac*I restriction sites of medium copy number vector, pACYCDuet-I (Novagen); Cm^R. ***Note:*** *caf* locus is under its own native regulatory system.	3, SM lab stock
**pACYC-R**	pACYC-F1 based plasmid contains *caf1R* under the control of *caf1R-caf1M* intergenic region; Cm^R.	This study
**pACYC-MA1**	pACYC-F1 based plasmid contain *cafMA1* operon under the control of *caf1R-caf1M* intergenic region; Cm^R.	This study
**pRS*caf1R′ -M′ -lacZ***	Contains *caf1R-caf1M* intergenic region (327 bp) plus first 192 and 158 bp (from the ATG start codon) of *caf1R* and *caf1M*. Whole fragment was cloned in between *Bam*HI and *Eco*RI sites of *lac-Z* transcriptional reporter, pRS550 plasmid (1); Amp^R.	Qahtani, A. and Pereira, I.; SM lab stock
**pDKG*caf1M′*$_{-197+158}$-*lacZ***	Derivative of pRS*caf1R′ -M′ -lacZ*, containing 197 bp upstream of the *caf1M* start codon. This fragment harbors R3′ and R4′ repeats and all predicted promoters for *cafMA1.*	This study

**pDKG*caf1M′*$_{-184+158}$-*lacZ***	Derivative of pRS*caf1R′-M′-lacZ* containing, 184 bp upstream of the *caf1M* start codon. This fragment possesses R4′ repeat and all predicted promoters for *cafMA1.*	This study
**pDKG*caf1M′*$_{-169+158}$-*lacZ***	Derivative of pRS*caf1R′-M′-lacZ* containing, 169 bp upstream of the *caf1M* start codon. This fragment possesses part of R4′ repeat and all predicted promoters for *cafMA1.*	This study
**pDKG*caf1M′*$_{-141+158}$-*lacZ***	Derivative of pRS*caf1R′-M′-lacZ* containing, 141 bp upstream of the *caf1M* start codon. This fragment possesses downstream fragment of -10 motif of $P_M{}^{DKG-2}$ promoter for *cafMA1.*	This study
**pDKG*caf1M′*$_{-102+158}$-*lacZ***	Derivative of pRS*caf1R′-M′-lacZ* containing, 102 bp upstream of the *caf1M* start codon. This fragment possesses previously predicted $P_M{}^{K1-2}$ promoters for *cafMA1.*	This study

Antibiotic resistance marker: Amp^R- Ampicillin, Cm^R- Chloramphenicol, Kan^R- Kanamycin

Table S3. *E. coli* strains used in this study.

Strain	Purpose and feature	Genotype	Source
TOP10	Expression studies for pBAD based plasmids. Keeps constant level of L-arabinose during growth.	F^- *mcr*A Δ(*mrr-hsd*RMS-*mcr*BC) ϕ80*lacZ*ΔM15 Δ*lac*X74 *rec*A1 *ara*D139 Δ(*ara-leu*)7697 *gal*U *gal*K *rps*L (Str^R) *end*A1 *nup*G λ-	Invitrogen, UK Cat. C4040-10
Stellar™	Transformation of InFusion mixture. Enhanced ability to repair nicks in the InFused DNA fragment.	*$F^–$ endA1 supE44 thi-1 recA1 relA1 gyrA96 phoA* Φ80d_*lac ZΔM15* Δ(*$lacZYA^-$ argF)U169* Δ(*mrr^- $hsdRMS^-$ mcrBC) ΔmcrA* $λ^–$	Clontech, UK Cat. 636763
BL21(DE3)	General expression strain of λDE3 lysogen contains a chromosomal copy of T7 RNAP gene under the control of *lacUV5* promoter.	$F^–$ *omp*T *gal dcm lon hsd*S_B (r_B^- m_B^-) λ(DE3)	ThermoFisher Scientific, UK Cat. C600003
STAR pLysS	BL21(DE3) derivative, provides enhanced mRNA stability with low protein background.	F^-*omp*T *hsd*SB (r_B^-, m_B^-) *gal dcm rne*131 (DE3) pLysS (Cm^R)	LifeTechnologies, UK Cat. C6020-03
K12 ER2508	K12 derivative with *lon^-* mutation. Deficient in major ATP-dependent proteases. Improves stability of heterologous proteins in cytoplasm.	F^- *ara-14 leuB6 fhuA2* Δ(*argF-lac*) U169 *lacY1 lon*::*miniTn*10 (Tet^R)*glnV44 galK2 rpsL20* (Str^R) *xyl-5 mtl-5Δ*(*malB*) *zjc*::Tn*5*(Kan^R) Δ(*mcrC-mrr*)$_{HB101}$	NEB, UK Cat. E4127
SHuffle® T7	K12 derivative. Expresses DsbC chaperone in cytoplasm that enhances cytoplasmic disulfide bonds formation.	F′ *lac, pro, $lacI^Q$ /Δ(ara-leu)7697 araD139 fhuA2 lacZ*::*T7 gene1*Δ(*phoA*)*PvuII phoR ahpC** *galE* (or U) *galK λatt*::pNEB3-r1-*cDsbC* ($Spec^R$, *$lacI^q$*) Δ*trxB rpsL150*(Str^R) Δ*gor* Δ(*malF*)*3*	NEB, UK Cat. C3026H

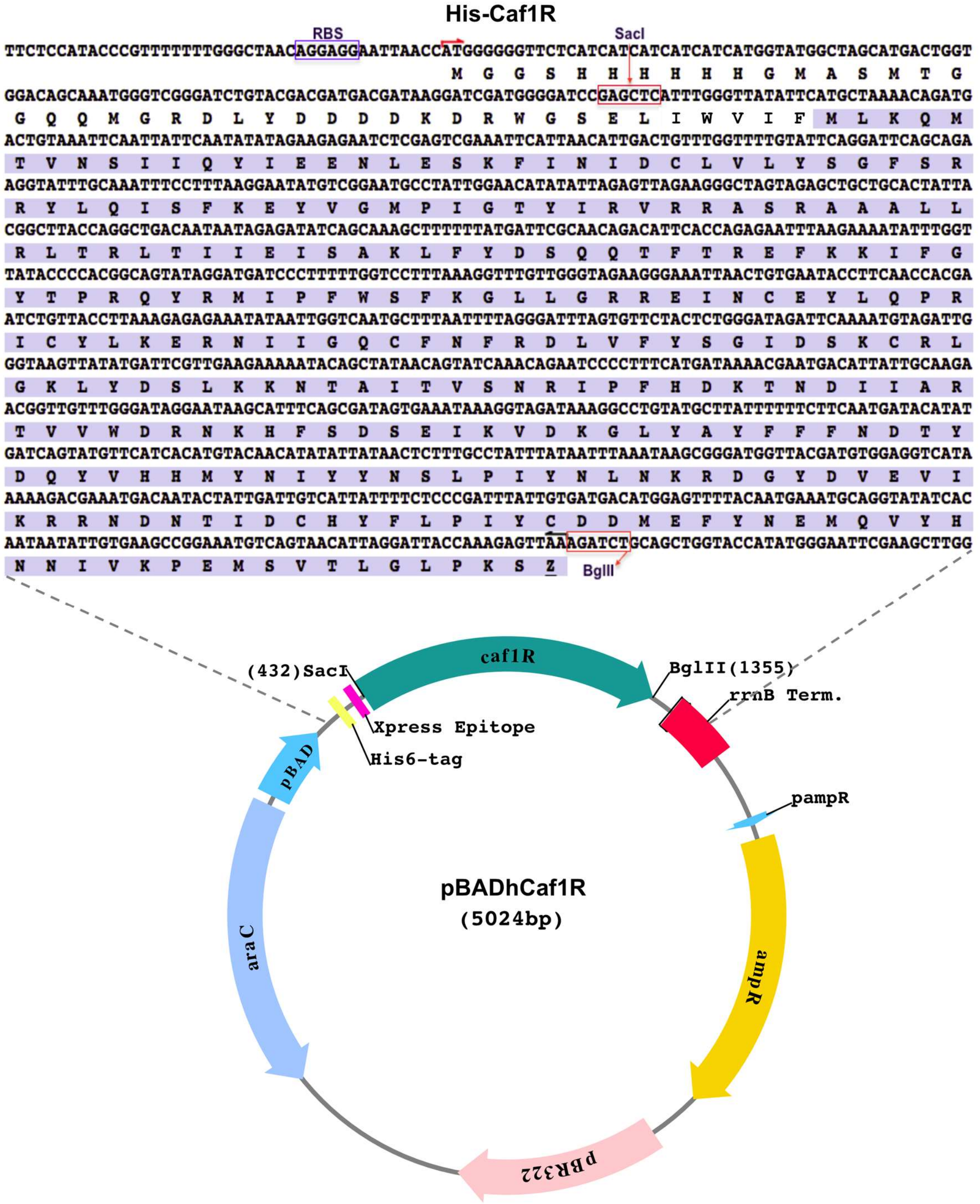

Figure S1. The pBADhCaf1R plasmid construct. Relevant DNA sequence of pBADhCaf1R. The gene encoding Caf1R was PCR-amplified from pFS2 plasmid [2,3] and cloned in between *Sac*I and *Bgl*II sites (red boxes) of the pBADHisA plasmid. Coding sequence derived from the native *caf1R* is highlighted in blue. Ribosomal binding site is enclosed in blue box. The translated sequence, His-Caf1R, has an N terminal addition of 6 His residues followed by Xpress epitope resulting in a 40.98 kDa protein. Expression is under control of the P_{BAD} promoter and induced by L-arabinose. Resistance marker is ampicillin. Circular plasmid map of the pBADhCaf1R with important features is indicated below.

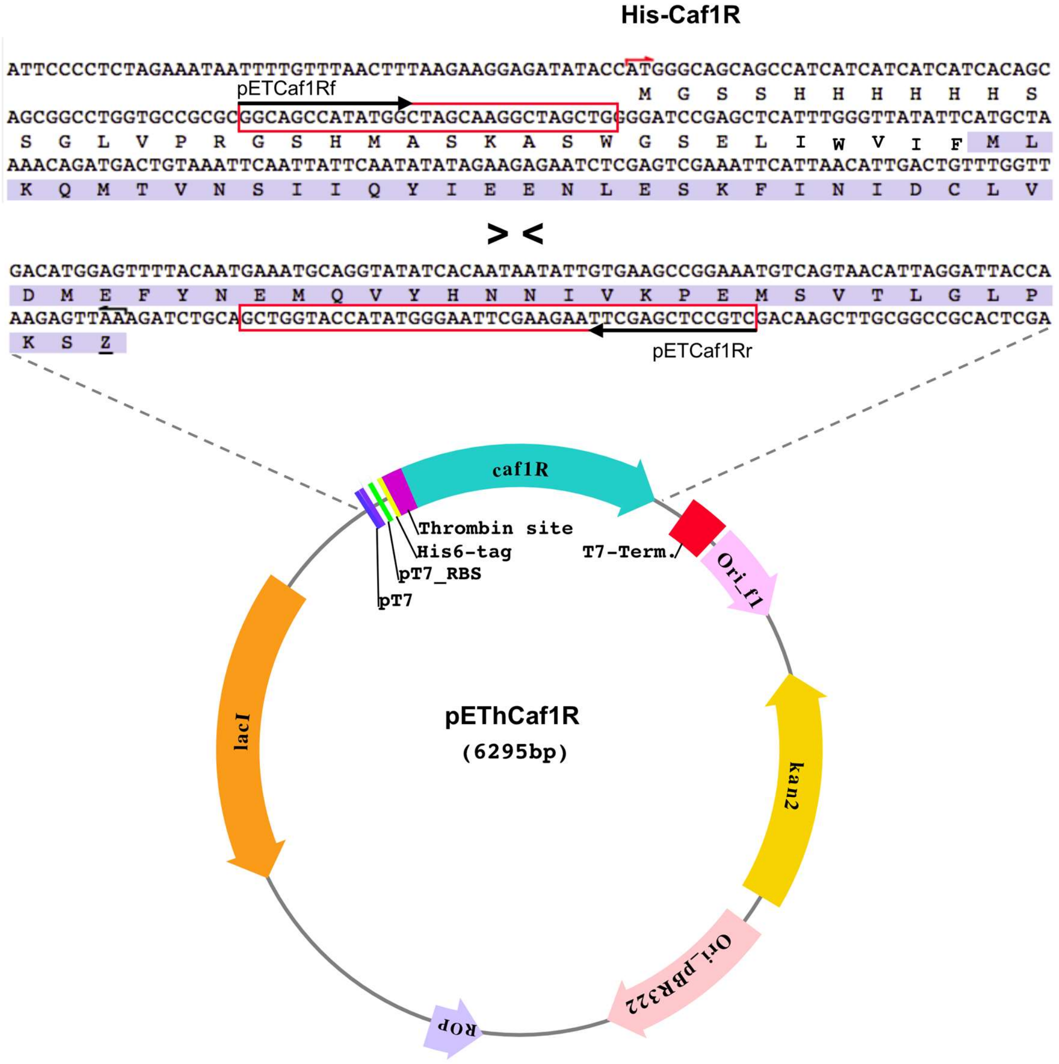

Figure S2. The pEThCaf1R plasmid construct. Relevant DNA sequence of pEThCaf1R, showing fusion joints and encoded His-Caf1R (338 aa; ~ 40 kDa), which has an N terminal addition of 6 His residues followed by a thrombin cleavage site. Encoded amino acids from *caf1R* are highlighted in blue. ><, represents sequence not shown. InFusion primers are enclosed in red boxes. Expression is under control of chromosomally encoded T7 polymerase, requires expression in *E. coli* BL21(DE3) and is induced by IPTG. Resistance marker is kanamycin. Circular plasmid map of pEThCaf1R with important features is indicated below.

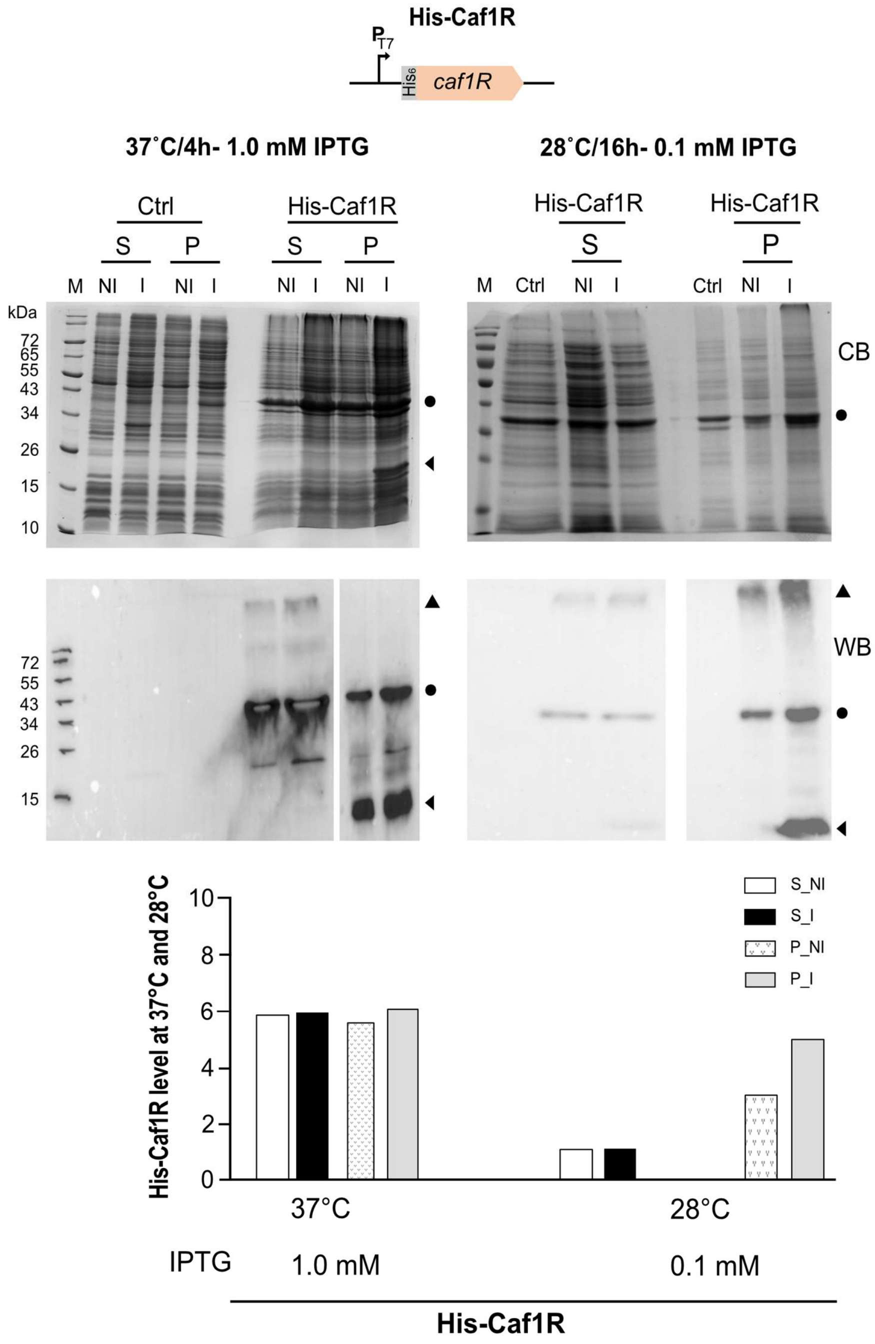

Figure S3. Expression of His-Caf1R (native *caf1R*) from *E. coli* BL21(DE3). Schematic of His-Caf1R from pEThCaf1R plasmid. CB-stained SDS-PAGE gel and WB analysis of full-length His-Caf1R expressed in *E. coli* BL21 (DE3). Total amount of full-length His-Caf1R was quantified from the respective WB. Cells were induced with IPTG at 37 °C and 28 °C, as indicated. Equal number of cells (1 OD unit) were harvested and mixed in 100 μL of PBS supplemented with 1× proteases inhibitor (EDTA-free) and lysed by sonication. Soluble supernatant (S) and insoluble pellet (P) fractions were prepared by ultracentrifugation of sonicated cell lysate. Expected location of His-Caf1R, confirmed in WB, is indicated by a dot (•). Truncated product is indicated by black arrow head. Location of high-molecular weight insoluble aggregates is represented by black triangle (Δ). Expression of His-Caf1R and degradation products were identified with antiHis-HRP monoclonal antibody and verified in comparison with negative control (Ctrl, empty plasmid). IPTG-induced and non-induced samples are indicated by I and NI, respectively. M, protein size (kDa) marker.

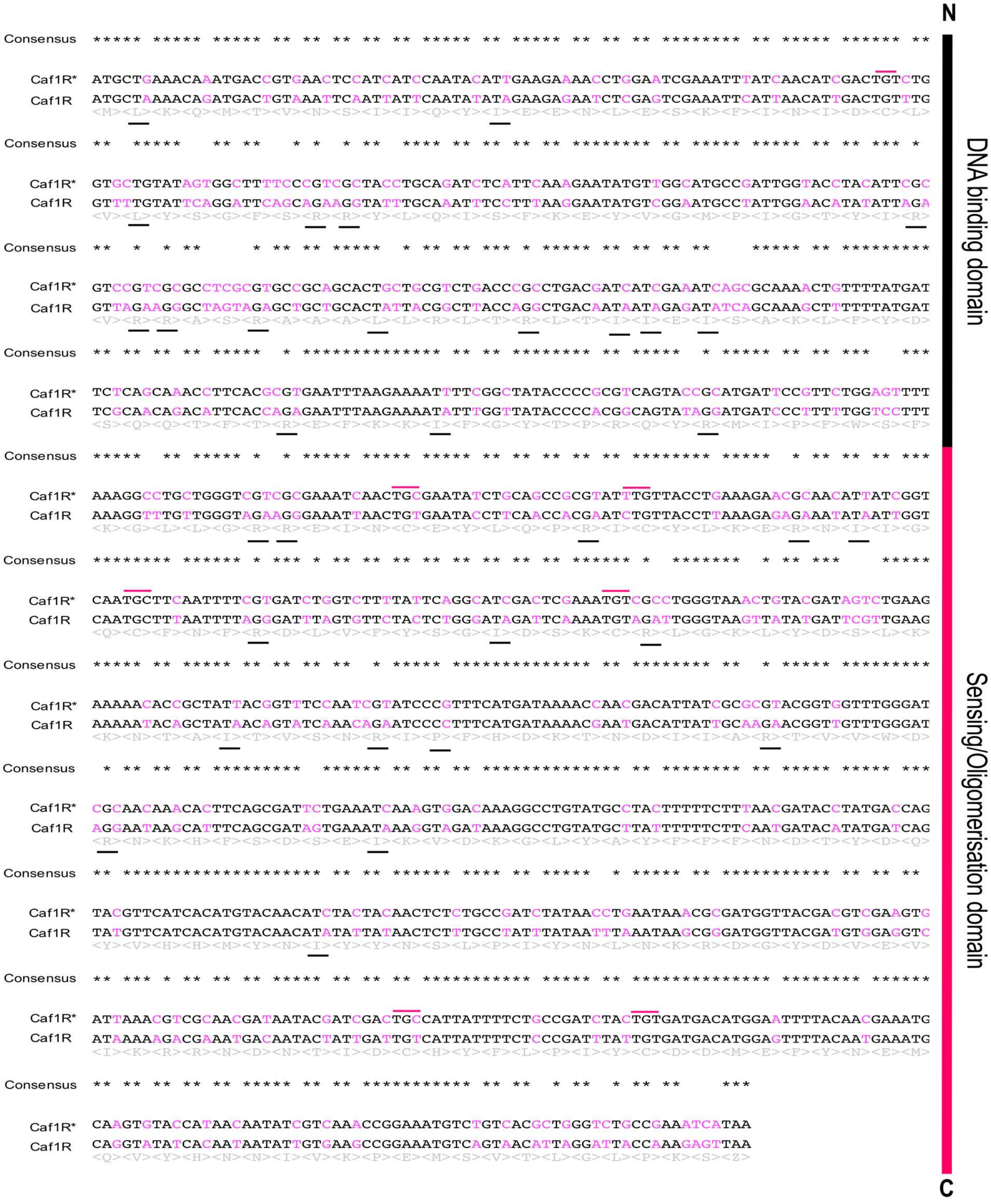

Figure S4. Sequence alignment of native Caf1R with codon-optimised Caf1R*. Rare codons identified by RaCC calculator are indicated by black underlines (32 amino acids). Changes built by GenScript, USA throughout *caf1R* ORF are indicated in pink (232 nucleotides encoding 162 amino acids). The 7 Cys residues are indicated by red line above each. Consensus nucleotides are indicated by*. Deduced amino acid sequence is indicated underneath of the alignment. Sequence encompassing the N-terminal DNA-binding domain (DBD) and C-terminal sensing/oligomerisation domain is indicated by respective thick vertical black and red line. Codon- optimised *caf1R** supplied as pEThCaf1R*.

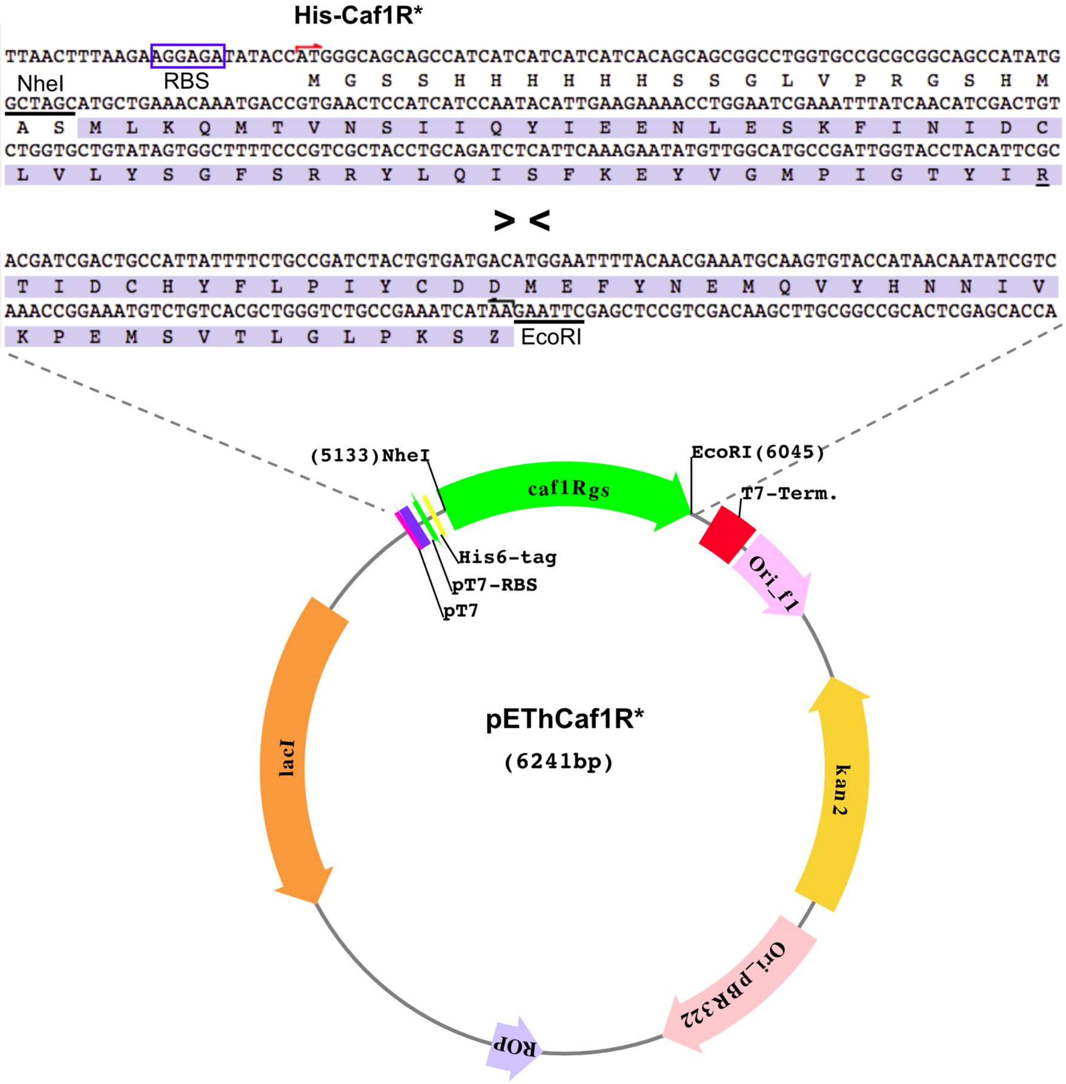

Figure S5. The pEThCaf1R* plasmid construct. Sequence of codon-optimised synthetic *caf1R* (*caf1R*gs) was cloned in between *Nhe*I and *Eco*RI sites (red boxes) of pET28a+. The encoded *caf1R* amino acid sequence is highlighted in blue with upstream fusion of His6-tag followed by a short linker to create His-Caf1R*, 325 aa, ~38.5 kDa). Ribosomal binding site, RBS is indicated in blue box. Expression is under control of chromosomally encoded T7 polymerase, requires expression in *E. coli* BL21(DE3) and is induced by IPTG. Resistance marker is kanamycin. Circular plasmid map of pEThCaf1R* with important features is indicated below.

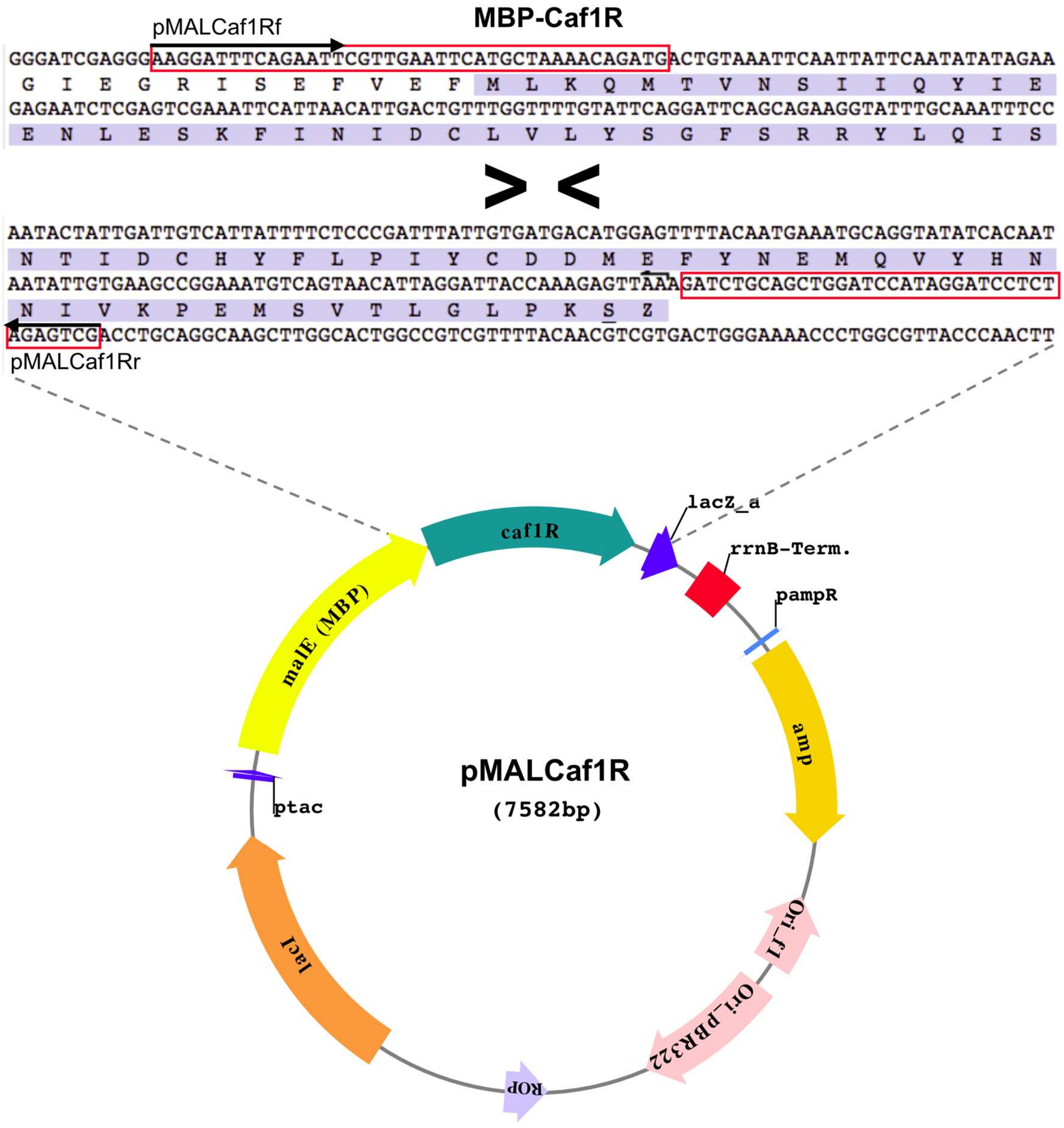

Figure S6. The pMALCaf1R plasmid construct. The pMALCaf1R encodes a MBP-Caf1R fusion (696 aa) of 79.36 kDa. Translated sequence derived from native *caf1R* (highlighted in blue); ><, represents sequence not shown; InFusion primers on either end (red box). Expression is under control of the P_{tac} promoter and is induced by IPTG. Resistance marker is ampicillin. Circular plasmid map of pMALCaf1R with important features is indicated below.

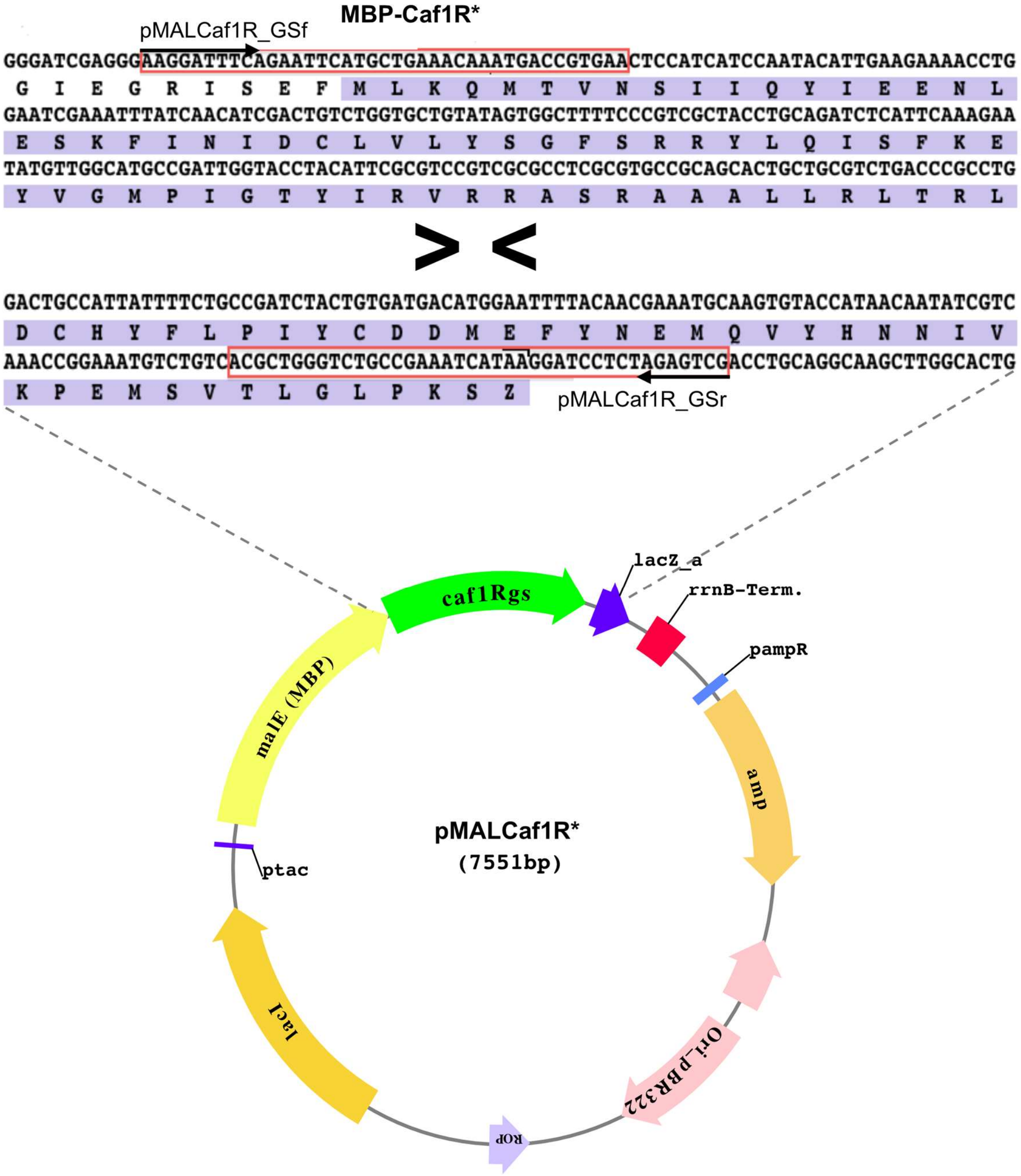

Figure S7. The pMALCaf1R* plasmid construct. The deduced amino acid sequence of the codon-optimised Caf1R is highlighted in blue, fused to N-terminus MBP (only a few linker residues (black) are shown). InFusion primers, used in PCR amplification of *caf1R** from pEThCaf1R*, are shown in red boxes. Complete fusion of MBP-Caf1R* codes 693 aa with a calculated molecular weight, 78.98 kDa. Expression is under control of the P_{tac} promoter and is induced by IPTG. Resistance marker is ampicillin. Circular plasmid map of pMALCaf1R* with important features is shown underneath.

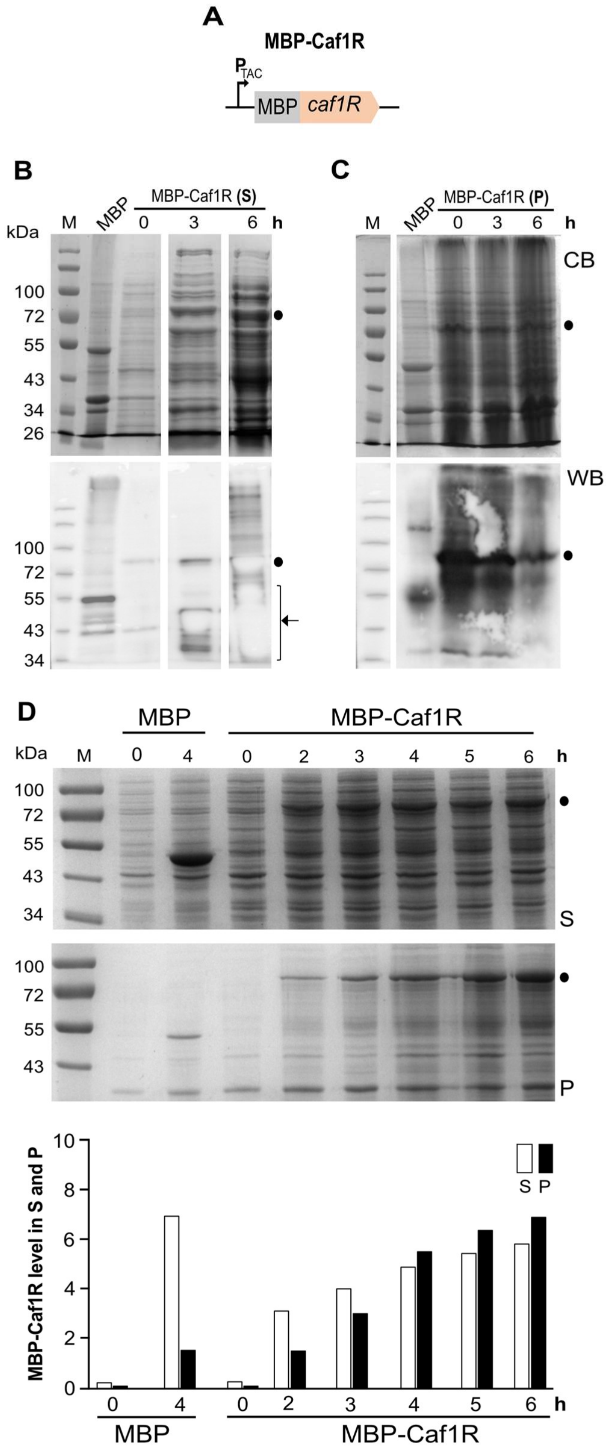

Figure S8. Expression of MBP-Caf1R (native *caf1R*) from *E. coli* BL21(DE3) and K12 ER2508. (A) Schematic of MBP-Caf1R from pMALCaf1R plasmid. MBP-Caf1R expression from *E. coli* BL21(DE3) **(B-C)**, and from *E. coli* K12 ER2508 **(D)**. As indicated, expression was monitored at 37 °C with 0.35 mM IPTG induction. Samples were processed for isolation of soluble (S) and insoluble (P) fractions at indicated times post IPTG-induction. Expected location of full-length MBP-Caf1R is indicated by a dot (•) and degradation/ truncation is shown by an arrow (⟵). Expression of MBP (Ctrl) from cells carrying empty plasmid (pMALc2x) can be seen at about 45 kDa. Relative level of MBP-Caf1R in S and P fractions was quantified from K12 ER2508 **(E).** Non-induced samples (0 h) and IPTG induced samples (2-6 h). M, protein size (kDa) marker.

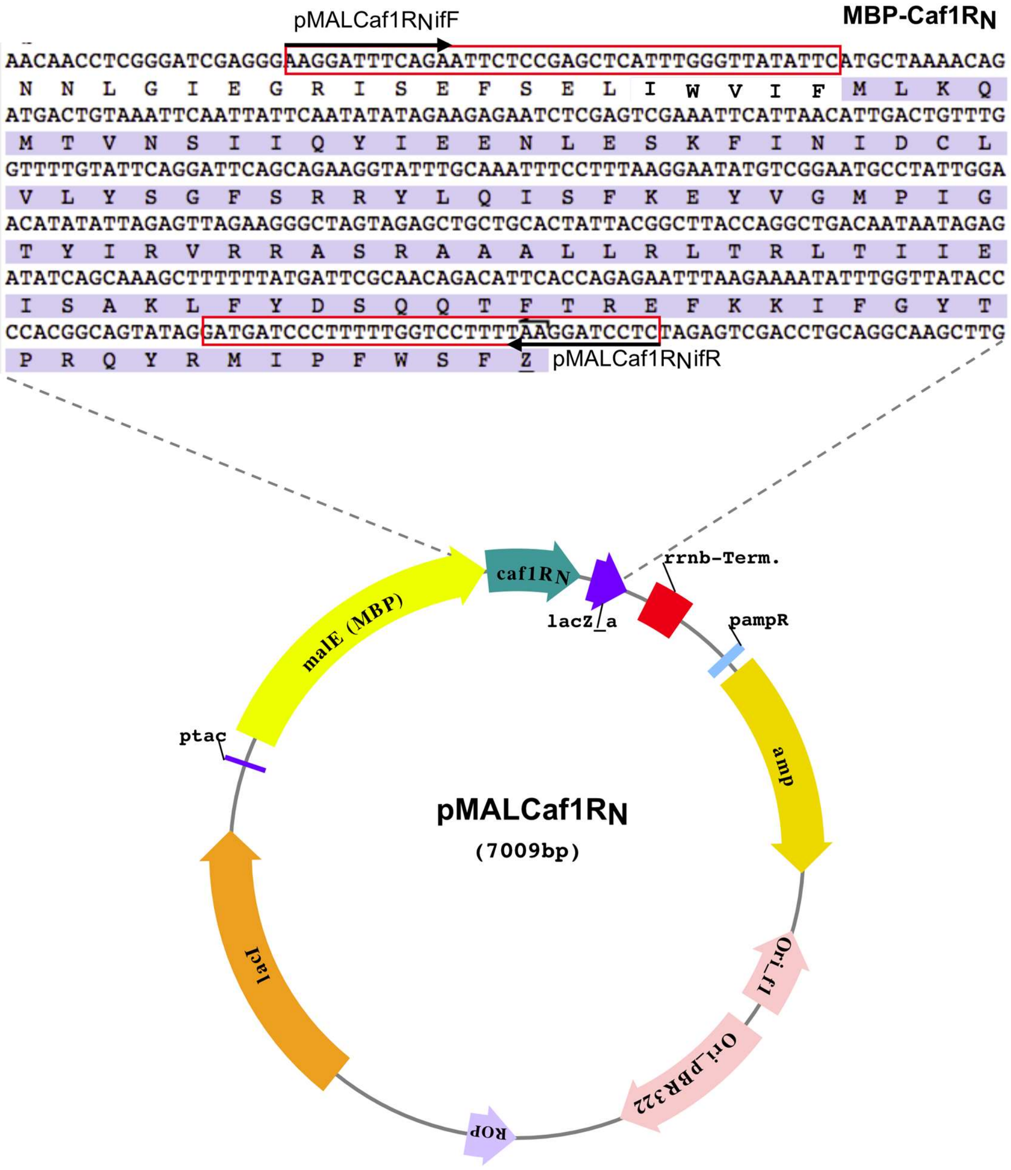

Figure S9. The pMALCaf1R$_N$ plasmid construct. The gene encoding Caf1R$_N$ was PCR-amplified from pMALCaf1R plasmid. The deduced amino acid sequence of the native Caf1R$_N$ is highlighted in blue, at the N-terminus this is fused to MBP (only a few linker residues (black) are shown). InFusion primers on either side are shown in red boxes. Complete fusion of MBP-Caf1R$_N$ codes 503 aa with a calculated molecular weight, 57.30 kDa. Expression is under control of the P$_{tac}$ promoter and is induced by IPTG. Resistance marker is ampicillin. Circular plasmid map of pMALCaf1R$_N$ with important features is shown underneath.

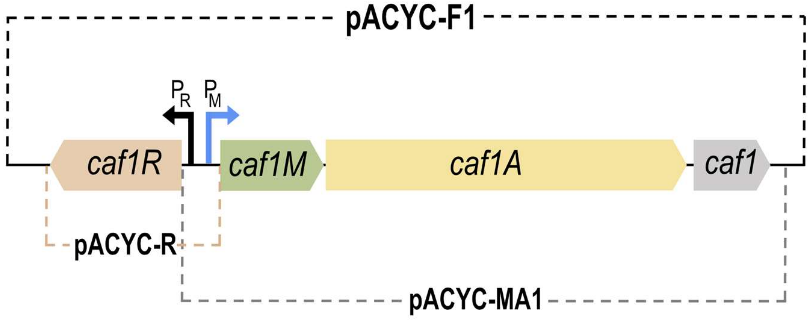

Figure S10. The pACYC-R and pACYC-MA1 plasmid constructs. Organisation of the *caf1* genes cluster (5.128 kb) cloned in the pACYCDuet-I vector and designed pACYC-F1 plasmid [**4**]. Genes are drawn as per their size and location, promoters transcribing *caf1R* (black arrow, P_R) and *cafMA1* **(blue arrow, P_M) are indicated. Both pACYC-R and pACYC-MA1 were designed by Inverse PCR from pACYC-F1 template using Q5 HiFi DNA polymerase (New England Biolabs, UK). Briefly, DNA fragment encompassing either full-length *caf1R* or *cafMA1* operon under the control of native *caf1R-caf1M* intergenic region was PCR-amplified using primers pair, pACYC*caf1R*f and pACYC*caf1R*r (for pACYC-R) or Inv*caf1R*for and Inv*caf1R*rev (for pACYC-MA1). Amplified-fragment containing desired region was gel-purified, digested either with *Sac*I (for pACYC-R) or *Nco*I (for pACYC-MA1) restriction enzymes and ligated by T4-DNA ligase (Thermo Fisher Scientific, UK). Both constructs were confirmed by sequencing. For all 3 plasmids induction is under control of the native *caf* regulatory region, at 37°C. Resistance marker is chloramphenicol.**

References

1. Simons, R.W.; Houman, F.; Kleckner, N. Improved single and multicopy *lac*-based cloning vectors for protein and operon fusions. *Gene.* **1987**, *53*, 85–96.
2. Galyov, E.; Karlishev, A.; Chernovskaya, T.; Dolgikh, D.; Smirnov, O.; Volkovoy, K.; Abramov, V.; Zav'yalov, V. Expression of the envelope antigen F1 of Yersinia pestis is mediated by the product of caf1M gene having homology with the chaperone protein PapD of Escherichia coli. *FEBS Lett.* **1991**, *286*, 79–82, doi:10.1016/0014-5793(91)80945-y
3. Karlyshev, A.V.; Galyov, E.E.; Abramov, V.M.; Zav'yalov, V.P. Caf1R gene and its role in the regulation of capsule formation of *Y. pestis*. *FEBS Lett.* **1992**, *305*, 37–40.
4. Kumar, D. Caf1R-Mediated Regulation of the F1 Surface Antigen of *Yersinia Pestis.* Ph.D. Thesis, University of Reading, Reading, UK, **2016**. British Library, UK (EthoS ID: 690097).
